# Supplementary material for: Confining single Er3+ ions in sub-3 nm NaYF4 nanoparticles to induce slow relaxation of the magnetisation
Source: Nat Commun. 2024 Apr 25;15:3498. doi: 10.1038/s41467-024-47682-x (PMC11045814; doi:10.1038/s41467-024-47682-x)
Supplement: Supplementary file 1 — Supplementary Information [file 41467_2024_47682_MOESM1_ESM.pdf]

Supplementary Information for

Confining single Er^{3+} ions in sub-3 nm $NaYF_4$ nanoparticles to induce slow relaxation of the magnetisation.

Diogo A. Gálico,[1] Emille M. Rodrigues,[1] Ilias Halimi,[1] Juho Toivola,[3] He Zhao[4], Jiahui Xu,[4], Jani O. Moilanen,[3*] Xiaogang Liu,[4] Eva Hemmer,[1,2*] Muralee Murugesu[1,2*]

[1] Department of Chemistry and Biomolecular Sciences, University of Ottawa, Ottawa, Ontario K1N 6N5, Canada

[2] Centre for Advanced Materials Research (CAMaR), University of Ottawa, Ottawa, Ontario K1N 6N5, Canada.

[3] Department of Chemistry, Nanoscience Centre, University of Jyväskylä, P.O. Box 35, FI-40014, Finland.

[4] Department of Chemistry, National University of Singapore, 3 Science Drive 3, 117543, Singapore.

*Corresponding Authors: M. Murugesu (E-mail: m.murugesu@uottawa.ca), E. Hemmer (ehemmer@uottawa.ca)

Table of Contents

1. Additional Experimental Details ... 2

2. Additional Characterization of α-$NaYF_4$:Er^{3+} (X %) ... 4

3. Monte Carlo Simulations ... 7

4. Additional Magnetic Data ... 9

5. Computational Results ... 23

1. Additional Experimental Details

Synthesis of α-NaYF$_4$:Er^{3+} (X %) nanoparticles

Supplementary Table 1: Overview of the $YCl_3·6H_2O$ and $ErCl_3·6H_2O$ precursor quantities used in the synthesis of the α-NaYF$_4$:Er (X %) nanoparticles (NPs), nominal and ICP results for the Er^{3+} dopant concentrations, and the resulting NP sizes based on TEM analysis.

Sample	Nominal Er^{3+} concentration (mol %)	YCl_3 $·6H_2O$ (mmol)	$ErCl_3$ $·6H_2O$ (mmol)	NP diameter (nm)	ICP Er^{3+} concentration (mol %)
NP1	1.0	0.6187	0.0063	2.7 ± 0.3	0.9
NP2	2.0	0.6125	0.0125	2.5 ± 0.3	2.0
NP4	4.0	0.6000	0.0250	2.5 ± 0.3	4.4
NP6	6.0	0.5874	0.0376	2.6 ± 0.3	6.1
NP8	8.0	0.5750	0.0500	2.5 ± 0.3	8.3
NP10	10.0	0.5625	0.0625	2.6 ± 0.3	10.1

Calculation of the number of Er^{3+} ions per nanoparticle

The volume of one NP (V_{NP}) was calculated using Equation (1) assuming a perfect spherical shape for the NP:

$$V_{NP} = \frac{4}{3}\left(\frac{d}{2}\right)^3 \pi \qquad (1)$$

where d is the mean diameter of each NP composition as obtained from the TEM size distributions given at Table S1 and Figure S2. The calculated NP volumes are given at Table S2.

The volume of the unit cell (V_{uc}) and the number of formula units (Z) of α-NaYF$_4$ was taken from the standard PDF card [00-006-0342]:

$$V_{uc} = 161.7 \text{ Å}^3 \text{ and } Z = 2$$

Therefore, the number of unit cells in each NP ($\#_{uc}$) was calculated by Equation (2):

$$\#_{uc} = \frac{V_{NP}}{V_{uc}} \qquad (2)$$

Knowing that each unit cell contains two RE^{3+} ions, the total number of RE ions ($\#_{RE/NP}$) in each NP was calculated by Equation (3) and is given at Table S2:

$$\#_{RE/NP} = 2 \times N_{uc} \qquad (3)$$

Assuming that the NP synthesis resulted in a statistical distribution of the Er^{3+} dopants in the α-$NaYF_4$ NPs, the number of Er^{3+} ions *per* NP ($\#_{Er/NP}$) for each Er^{3+} dopant concentration ($\#_{Er\%}$) can be calculated by Equation (4):

$$\#_{Er/NP} = \frac{\#_{RE/NP} \times \#_{Er\%}}{100} \quad (4)$$

The resultant numbers of Er^{3+} ions *per* NP as a function of the Er^{3+} dopant concentration are summarized in Table S2.

Supplementary Table 2: Nanoparticle diameter obtained from TEM size distributions (*d*), calculated nanoparticle volume (V_{NP}), number of RE^{3+} ions *per* NP ($\#_{RE/NP}$), and number of Er^{3+} ions *per* NP ($\#_{Er/NP}$) for each Er^{3+} dopant concentration.

Sample	*d* (Å)	V_{NP} (Å^3)	$\#_{RE/NP}$	$\#_{Er/NP}$
NP1	26.9 (0.4)	10187 (454)	126 (6)	1.1 (0.1)
NP2	25.2 (0.3)	8375 (299)	104 (4)	2.1 (0.1)
NP4	24.7 (0.3)	7886 (287)	98 (4)	4.3 (0.2)
NP6	26.2 (0.3)	9412 (323)	116 (4)	7.1 (0.2)
NP8	25.1 (0.3)	8276 (297)	102 (4)	8.5 (0.3)
NP10	26.3 (0.3)	9520 (326)	118 (4)	11.9 (0.3)

2. Additional Characterization of α-$NaYF_4$:Er^{3+} (X %)

Crystalline phase

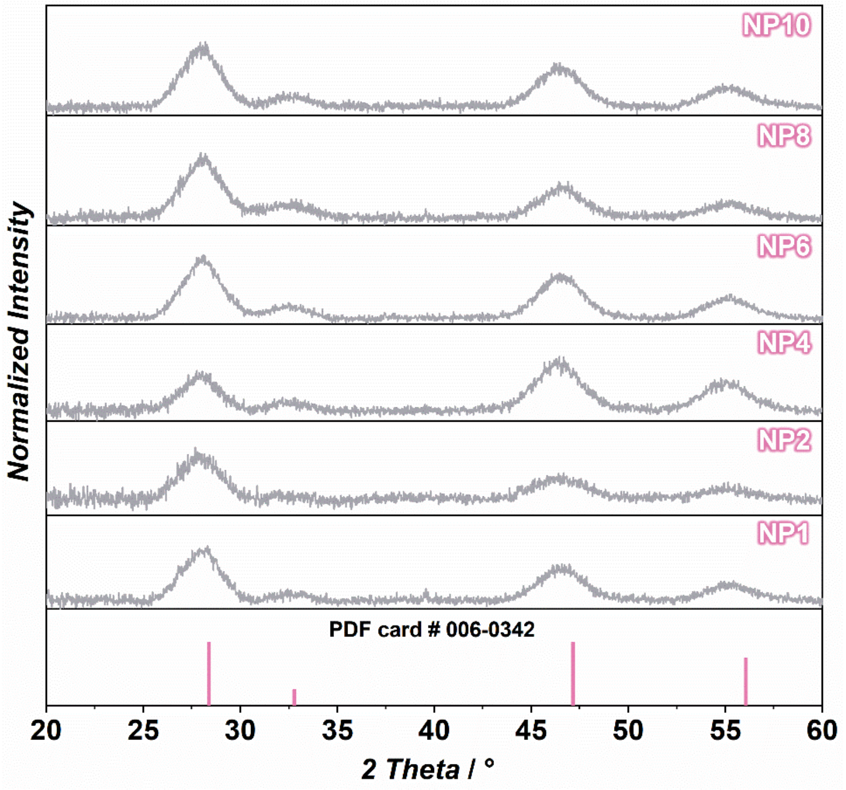

Supplementary Figure 1: Crystalline phase analyze *via* powder XRD: XRD patterns of cubic-phase α-$NaYF_4$:Er^{3+} NPs obtained by microwave-assisted thermal decomposition. Reference: α-$NaYF_4$, PDF card [00-006-0342].

Size and size distribution

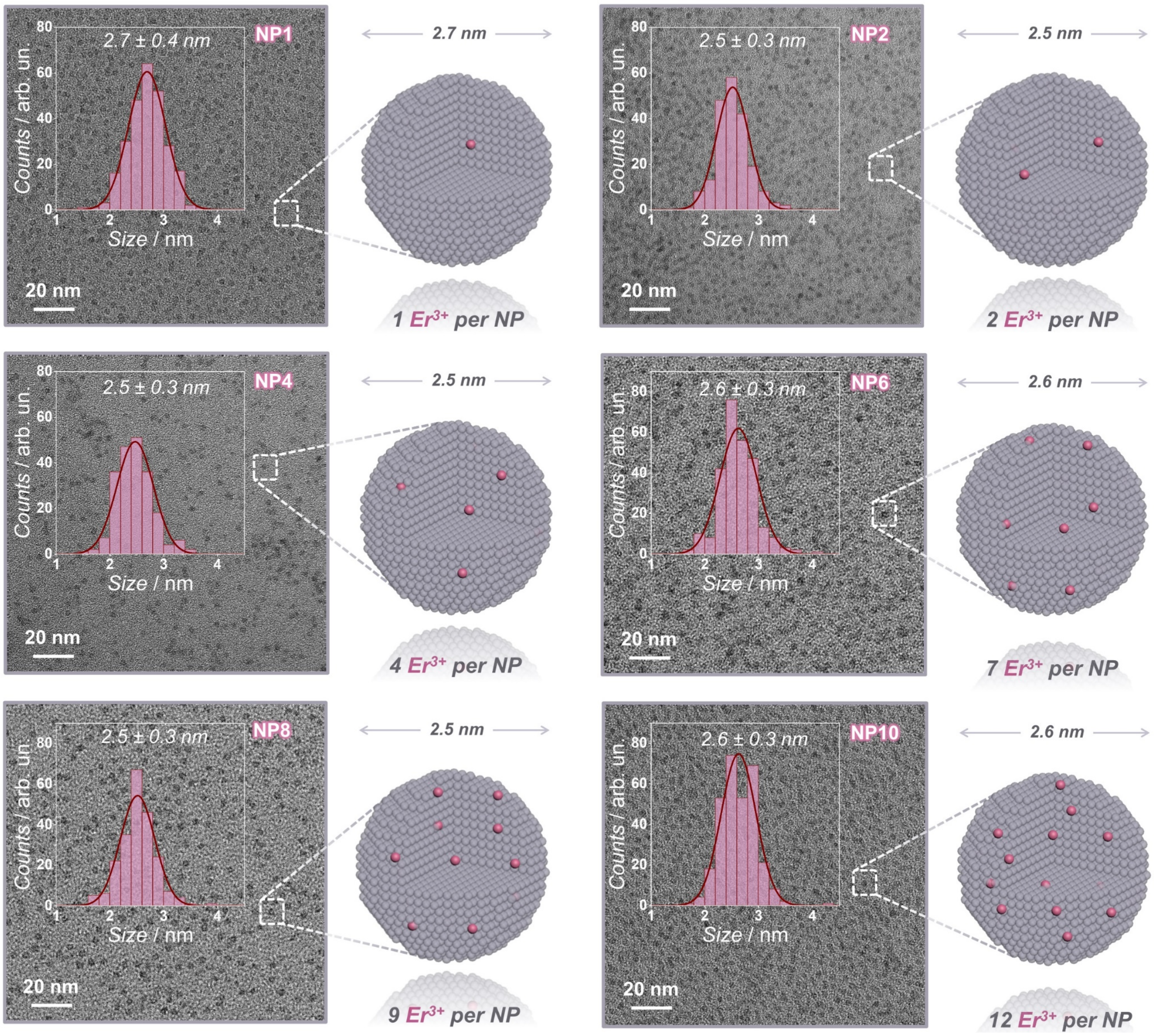

Supplementary Figure 2: Transmission electron microscopy for the synthesized NPs. TEM images and size distributions of the oleate-capped α-$NaYF_4$:Er^{3+} (X %) NPs obtained by microwave-assisted thermal decomposition. The schemes graphically represent the number of Er^{3+} ions per NP for each of the NP compositions.

Thermogravimetric analysis (TGA)

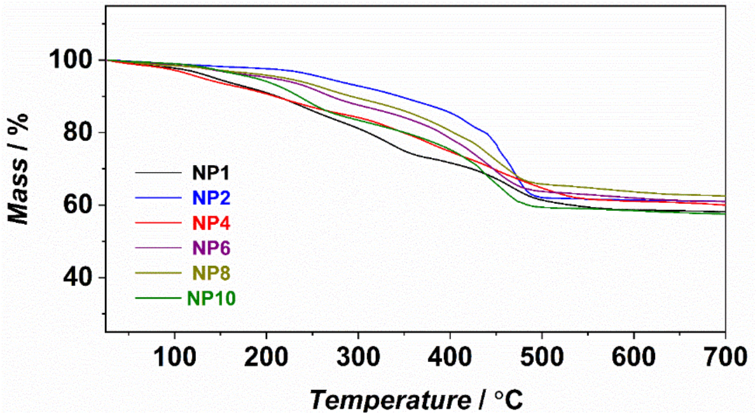

Supplementary Figure 3: Thermal analyses for the synthesized NPs. TGA curves for NP1 (5.234 mg), NP2 (11.721 mg), NP4 (6.012 mg), NP6 (9.021 mg), NP8 (9.222 mg), and NP10 (6.342 mg). These data indicate a similar amount of residual hexane and oleate ligands on the surface of the synthesized NPs. Mass loss below 200 °C is due to residual hexane used as solvent for NP dispersions. Above this temperature, mass loss is due to the surface oleate groups.

3. Monte Carlo Simulations

The average distance (*D*) between adjacent Er^{3+} ions in the particle was calculated using MATLAB software based on C+ language. The model was established based on the assumption of uniform distributions of dopant (Er^{3+}) ions within cubic Y^{3+}-based $NaYF_4$ host particles. The calculation was repeated for 10000 times with Monte Carlo Simulink.

The diameter of the particle was determined by TEM analysis (Figure S2). The number of Er^{3+} ions was obtained by ICP analysis. Taking into account these experimental data, the number of Y and Na ions, respectively, in each particle was calculated based on cubic-phase $NaYF_4$ unit cell dimensions. The condition "# RE ions = # Na ions" was applied. This resulted in a minor deviation from experimental (ICP) results for the number of Y ions, which may be the result of experimental error/inaccuracy in size determination and ICP.

Supplementary Table 3: Number of Y and Na ions and the average Er^{3+}-Er^{3+} distances (*D*) obtained *via* Monte Carlo simulations (values in blue are calculated data; values in black are experimental data).

Sample	*d* (Å)	# Er^{3+} per NP †	# RE^{3+} per NP †	# Y^{3+} per NP	# Na^{+} per NP ‡	*D* (Å)
NP1	26.9	1	126	123	125	-
NP2	25.2	2	104	98	101	12.92
NP4	24.7	4	98	96	101	9.11
NP6	26.2	7	116	105	113	7.44
NP8	25.1	9	102	91	101	6.52
NP10	26.3	12	118	100	113	6.08

† Data based on ICP analysis. Number of RE = ICP number of Er + ICP number of Y.
‡ Calculated number Na per NP. The total calculated numbers of the atoms were kept to be odd numbers, such that the numbers of [Y+Er] would be always one less than that of Na.

Figure S4 shows the possible distribution of Y^{3+} and Er^{3+} ions within one single particle as well as the Er^{3+}-Er^{3+} distance distribution as a function of the Er^{3+} dopant concentration. Only one of the possible distributions is presented as an example for each case, yet, the final distance values were calculated based on a 10000 times repetition.

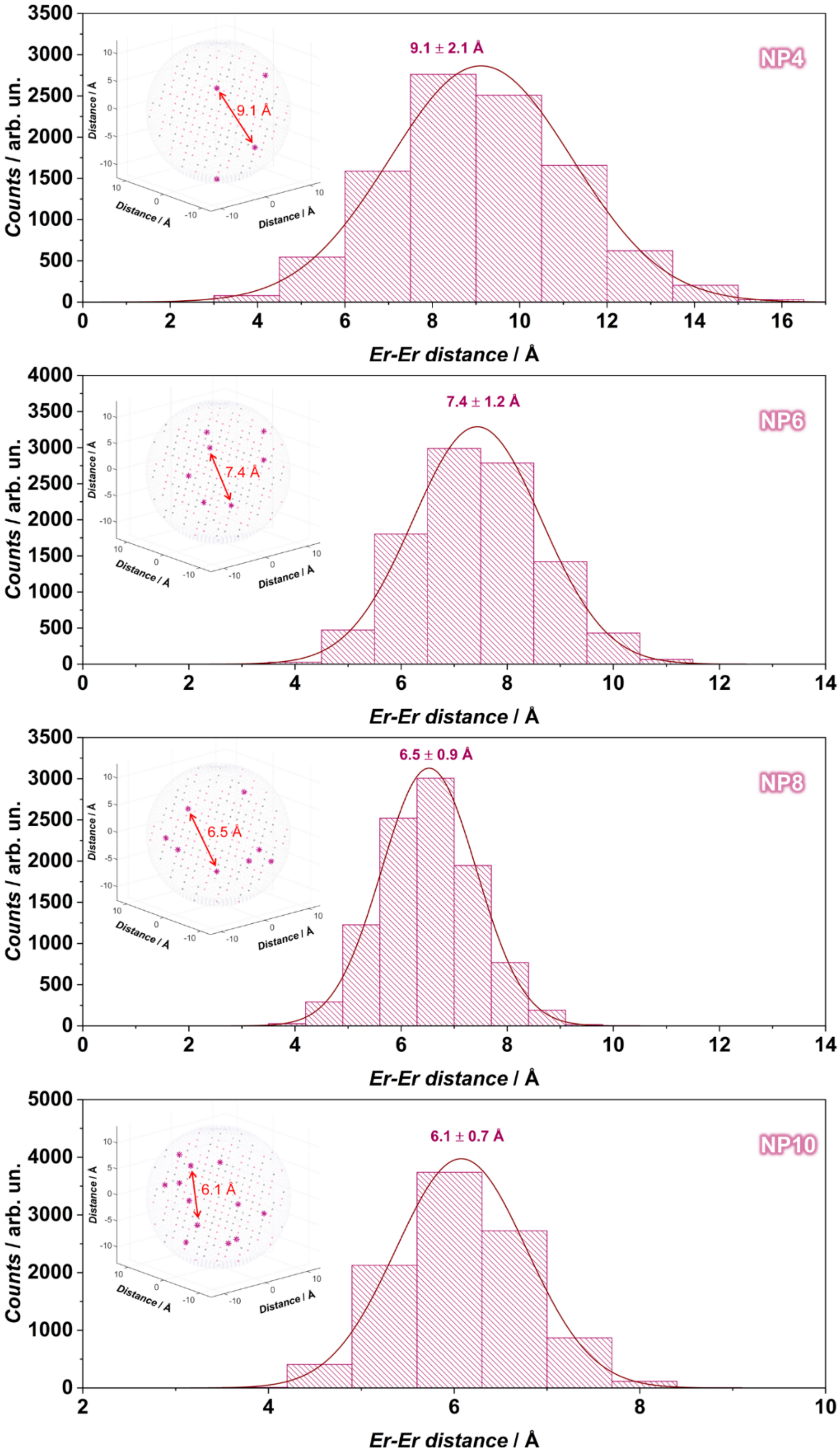

Supplementary Figure 4: Monte Carlo simulation for the synthesized NPs. Er^{3+}-Er^{3+} distance distributions for NP4, NP6, NP8, and NP10. Inset: 3D models obtained through Monte Carlo simulation displaying one possible scenario of the Er^{3+} ion distribution for each dopant concentration. The mean Er^{3+}-Er^{3+} distances for each of the NP compositions – based on a 10000 times repetition – are also shown.

4. Additional Magnetic Data

Supplementary Table 4: Best-fit parameters to the generalized and double Debye model for the field dependence of the the out-of-phase (χ'') magnetic susceptibility for **NP10**. Data collected at 1.9 K (Fig. 3).

H / Oe	χ_{s1}	χ_{t1}	α_1	τ_1	χ_{s2}	χ_{t2}	α_2	τ_2
400	-	-	-	-	0.73998	0.74001	0.008	1.15 x 10^{-1}
500	-	-	-	-	0.73999	0.74001	0.007	1.33 x 10^{-1}
600	-	-	-	-	0.73998	0.74002	0	1.22 x 10^{-1}
800	-	-	-	-	0.73996	0.74004	0.054	1.50 x 10^{-1}
1000	-	-	-	-	0.73995	0.74005	0.049	1.42 x 10^{-1}
1200	-	-	-	-	0.73986	0.74014	0.263	1.49 x 10^{-1}
1400	-	-	-	-	0.73991	0.74009	0.031	1.65 x 10^{-1}
1600	-	-	-	-	0.7399	0.7401	0	1.61 x 10^{-1}
1800	-	-	-	-	0.7399	0.7401	0.111	1.73 x 10^{-1}
2000	-	-	-	-	0.73987	0.74013	0.027	1.95 x 10^{-1}
2200	-	-	-	-	-	-	-	1.69 x 10^{-1}
2400	-	-	-	-	-	-	-	2.06 x 10^{-1}
2600	-	-	-	-	-	-	-	1.99 x 10^{-1}
2800	-	-	-	-	0.74976	0.75029	0.292	2.19 x 10^{-1}
3000	-	-	-	-	-	-	-	1.97 x 10^{-1}
3500	-	-	-	-	-	-	-	2.27 x 10^{-1}
4000	-	-	-	-	-	-	-	2.39 x 10^{-1}
4500	-	-	-	-	0.74981	0.75027	0.185	2.99 x 10^{-1}
5000	-	-	-	-	0.74989	0.75019	0.030	2.47 x 10^{-1}

Supplementary Table 5: Best-fit parameters to the generalized and double Debye model for the field dependence of the the out-of-phase (χ'') magnetic susceptibility for **NP8**. Data collected at 1.9 K (Fig. 3).

***H* / Oe**	χ_{s1}	χ_{t1}	α_1	τ_1	χ_{s2}	χ_{t2}	α_2	τ_2
800	-	-	-	-	0.75001	0.75007	0.339	9.97×10^{-2}
1000	-	-	-	-	0.75	0.75009	0.296	1.08×10^{-1}
1200	-	-	-	-	0.74998	0.7501	0.276	1.14×10^{-1}
1400	-	-	-	-	0.74997	0.75012	0.287	1.23×10^{-1}
1600	-	-	-	-	0.74995	0.75013	0.279	1.34×10^{-1}
1800	-	-	-	-	0.74994	0.75015	0.254	1.41×10^{-1}
2000	-	-	-	-	0.74993	0.75015	0.192	1.63×10^{-1}
2200	-	-	-	-	0.74992	0.75016	0.167	1.79×10^{-1}
2400	-	-	-	-	0.74991	0.75017	0.193	1.82×10^{-1}
2600	-	-	-	-	0.7499	0.75018	0.211	2.10×10^{-1}
2800	-	-	-	-	0.7499	0.75019	0.196	1.96×10^{-1}
3000	-	-	-	-	0.7499	0.75018	0.146	2.20×10^{-1}
3500	-	-	-	-	0.74989	0.75019	0.151	2.38×10^{-1}
4000	-	-	-	-	0.7499	0.75019	0.144	2.65×10^{-1}
4500	-	-	-	-	0.7499	0.75018	0.146	3.19×10^{-1}
5000	-	-	-	-	0.74991	0.75017	0.137	3.10×10^{-1}

Supplementary Table 6: Best-fit parameters to the generalized and double Debye model for the field dependence of the the out-of-phase (χ'') magnetic susceptibility for **NP6**. Data collected at 1.9 K (Fig. 3).

***H* / Oe**	χ_{s1}	χ_{t1}	α_1	τ_1	χ_{s2}	χ_{t2}	α_2	τ_2
800	-	-	-	-	0.75002	0.75007	0.198	9.66×10^{-2}
1000	-	-	-	-	0.75	0.75008	0.151	1.01×10^{-1}
1200	-	-	-	-	0.74999	0.7501	0.248	9.35×10^{-2}
1400	-	-	-	-	0.74998	0.7501	0.153	1.16×10^{-1}
1600	-	-	-	-	0.74997	0.75012	0.155	1.26×10^{-1}
1800	-	-	-	-	0.74995	0.75013	0.158	1.35×10^{-1}
2000	-	-	-	-	0.74995	0.75014	0.136	1.45×10^{-1}
2200	-	-	-	-	0.74994	0.75015	0.138	1.56×10^{-1}
2400	-	-	-	-	0.74994	0.75015	0.094	1.74×10^{-1}
2600	-	-	-	-	0.74993	0.75016	0.128	1.76×10^{-1}
2800	-	-	-	-	0.74992	0.75016	0.123	1.84×10^{-1}
3000	-	-	-	-	0.74991	0.75017	0.158	1.85×10^{-1}
3500	-	-	-	-	0.74991	0.75017	0.138	2.09×10^{-1}
4000	-	-	-	-	0.74992	0.75016	0.110	2.35×10^{-1}
4500	-	-	-	-	0.74992	0.75016	0.118	2.49×10^{-1}
5000	-	-	-	-	0.74993	0.75015	0.090	2.68×10^{-1}

Supplementary Table 7: Best-fit parameters to the generalized and double Debye model for the field dependence of the the out-of-phase (χ'') magnetic susceptibility for **NP4**. Data collected at 1.9 K (Fig. 3).

***H* / Oe**	χ_{s1}	χ_{t1}	α_1	τ_1	χ_{s2}	χ_{t2}	α_2	τ_2
200	0.75003	0.75009	0.231	4.42×10^{-5}	-	-	-	-
300	0.74989	0.75012	0.249	6.90×10^{-5}	-	-	-	-
400	0.74982	0.75016	0.233	1.03×10^{-4}	-	-	-	-
500	0.74992	0.75019	0.249	1.17×10^{-4}	-	-	-	-
600	0.74978	0.75021	0.254	1.33×10^{-4}	-	-	-	-
800	0.74977	0.75025	0.310	1.53×10^{-4}	-	-	-	-
1000	0.74483	0.74519	0.212	1.76×10^{-4}	0.73997	0.74003	0.328	5.72×10^{-2}
1200	0.74472	0.7452	0.224	1.81×10^{-4}	0.73997	0.74003	0.282	5.62×10^{-2}
1400	0.74477	0.74522	0.287	1.89×10^{-4}	0.73994	0.74006	0.065	6.30×10^{-2}
1600	0.74475	0.74521	0.262	2.02×10^{-4}	0.73995	0.74005	0.212	7.37×10^{-2}
1800	0.74479	0.74521	0.315	1.97×10^{-4}	0.73995	0.74005	0.134	8.77×10^{-2}
2000	0.74482	0.7452	0.318	1.58×10^{-4}	0.73995	0.74005	0.148	9.03×10^{-2}
2200	0.74461	0.74522	0.388	1.46×10^{-4}	0.73995	0.74005	0.091	1.08×10^{-1}
2400	0.74479	0.74521	0.414	1.40×10^{-4}	0.73994	0.74006	0.079	1.11×10^{-1}
2600	0.74478	0.74522	0.458	1.20×10^{-4}	0.73994	0.74006	0.087	1.21×10^{-1}
2800	0.74485	0.7452	0.464	9.52×10^{-5}	0.73994	0.74006	0.073	1.18×10^{-1}
3000	0.74464	0.74524	0.579	7.93×10^{-5}	0.73994	0.74006	0.061	1.31×10^{-1}
3500	0.74476	0.74525	0.562	5.00×10^{-5}	0.73994	0.74006	0.006	1.48×10^{-1}
4000	-	-	-	-	0.73993	7.40E-01	0.093	1.44×10^{-1}
4500	-	-	-	-	0.73995	0.74005	0.012	1.74×10^{-1}

Supplementary Table 8: Best-fit parameters to the generalized and double Debye model for the field dependence of the the out-of-phase (χ'') magnetic susceptibility for **NP2**. Data collected at 1.9 K (Fig. 3).

H / Oe	χ_{s1}	χ_{t1}	α_1	τ_1	χ_{s2}	χ_{t2}	α_2	τ_2
100	0.75001	0.75008	0.368	4.59×10^{-5}	-	-	-	-
200	0.74998	0.7501	0.343	1.07×10^{-4}	-	-	-	-
300	0.74997	0.75011	0.297	1.93×10^{-4}	-	-	-	-
400	0.74996	0.75012	0.280	2.41×10^{-4}	-	-	-	-
500	0.74996	0.75013	0.243	3.32×10^{-4}	-	-	-	-
600	0.74995	0.75013	0.241	3.89×10^{-4}	-	-	-	-
800	0.74994	0.75014	0.244	5.18×10^{-4}	-	-	-	-
1000	0.74993	0.75013	0.248	6.12×10^{-4}	-	-	-	-
1200	0.74491	0.74509	0.192	6.71×10^{-4}	0.73999	0.74001	0.321	5.93×10^{-2}
1400	0.74491	0.74509	0.186	6.91×10^{-4}	0.73998	0.74002	0.322	5.28×10^{-2}
1600	0.74491	0.74509	0.210	7.06×10^{-4}	0.73998	0.74002	0.318	6.04×10^{-2}
1800	0.7449	0.7451	0.275	7.43×10^{-4}	0.73999	0.74001	0.167	1.02×10^{-1}
2000	0.74491	0.74509	0.217	7.84×10^{-4}	0.73998	0.74002	0.245	8.76×10^{-2}
2200	0.74491	0.74509	0.220	7.26×10^{-4}	0.73998	0.74002	0.246	8.87×10^{-2}
2400	0.74492	0.74508	0.169	6.90×10^{-4}	0.73997	0.74003	0.324	6.62×10^{-2}
2600	0.74492	0.74508	0.222	6.92×10^{-4}	0.73998	0.74002	0.205	1.04×10^{-1}
2800	0.74492	0.74508	0.241	5.75×10^{-4}	0.73998	0.74002	0.178	1.03×10^{-1}
3000	0.74492	0.74508	0.313	5.23×10^{-4}	0.73998	0.74002	0.045	1.25×10^{-1}
3500	0.74492	0.74508	0.358	3.53×10^{-4}	0.73998	0.74002	0.069	1.43×10^{-1}
4000	0.74493	0.74507	0.410	2.48×10^{-4}	0.73998	0.74002	0.032	1.54×10^{-1}
4500	0.74492	0.74508	0.518	1.55×10^{-4}	0.73999	0.74001	0.012	1.72×10^{-1}
5000	0.74494	0.74506	0.534	1.09×10^{-4}	0.73999	0.74001	0.006	1.75×10^{-1}

Supplementary Table 9: Best-fit parameters to the generalized and double Debye model for the field dependence of the the out-of-phase (χ'') magnetic susceptibility for **NP1**. Data collected at 1.9 K (Fig. 3).

H / Oe	χ_{s1}	χ_{t1}	α_1	τ_1	χ_{s2}	χ_{t2}	α_2	τ_2
100	0.75001	0.75007	0.388	1.02×10^{-4}	-	-	-	-
200	0.75	0.75008	0.388	2.04×10^{-4}	-	-	-	-
300	0.75	0.75008	0.377	3.39×10^{-4}	-	-	-	-
400	0.75	0.75008	0.322	6.28×10^{-4}	-	-	-	-
500	0.74999	0.75009	0.357	6.99×10^{-4}	-	-	-	-
600	0.74999	0.75009	0.349	9.24×10^{-4}	-	-	-	-
800	0.74999	0.75009	0.332	1.33×10^{-3}	-	-	-	-
1000	0.74496	0.74504	0.249	1.42×10^{-3}	0.73999	0.74001	0.423	5.49×10^{-2}
1200	0.74496	0.74504	0.262	1.70×10^{-3}	0.74	0.74	0.150	5.40×10^{-2}
1400	0.74496	0.74504	0.243	1.68×10^{-3}	0.73999	0.74001	0.336	5.84×10^{-2}
1600	0.74496	0.74504	0.259	1.89×10^{-3}	0.73999	0.74001	0.303	1.08×10^{-1}
1800	0.74496	0.74504	0.184	1.83×10^{-3}	0.73999	0.74001	0.266	7.79×10^{-1}
2000	0.74496	0.74504	0.251	1.68×10^{-3}	0.73999	0.74001	0.248	8.10×10^{-2}
2200	0.74496	0.74504	0.213	1.68×10^{-3}	0.73999	0.74001	0.219	9.85×10^{-2}
2400	0.74497	0.74503	0.203	1.46×10^{-3}	0.73999	0.74001	0.278	7.45×10^{-2}
2600	0.74496	0.74504	0.252	1.44×10^{-3}	0.73999	0.74001	0.093	1.21×10^{-1}
2800	0.74496	0.74504	0.299	1.17×10^{-3}	0.73999	0.74001	0.131	1.11×10^{-1}
3000	0.74496	0.74504	0.319	9.02×10^{-4}	0.73999	0.74001	0.146	1.15×10^{-1}
3500	0.74497	0.74503	0.372	7.69×10^{-4}	0.73999	0.74001	0.125	1.34×10^{-1}
4000	0.74497	0.74503	0.340	4.32×10^{-4}	0.73999	0.74001	0.198	1.21×10^{-1}
4500	0.74497	0.74503	0.539	3.12×10^{-4}	0.74	0.74	0.175	1.53×10^{-1}
5000	0.74497	0.74503	0.599	2.14×10^{-4}	0.74	0.74	0.212	1.73×10^{-1}

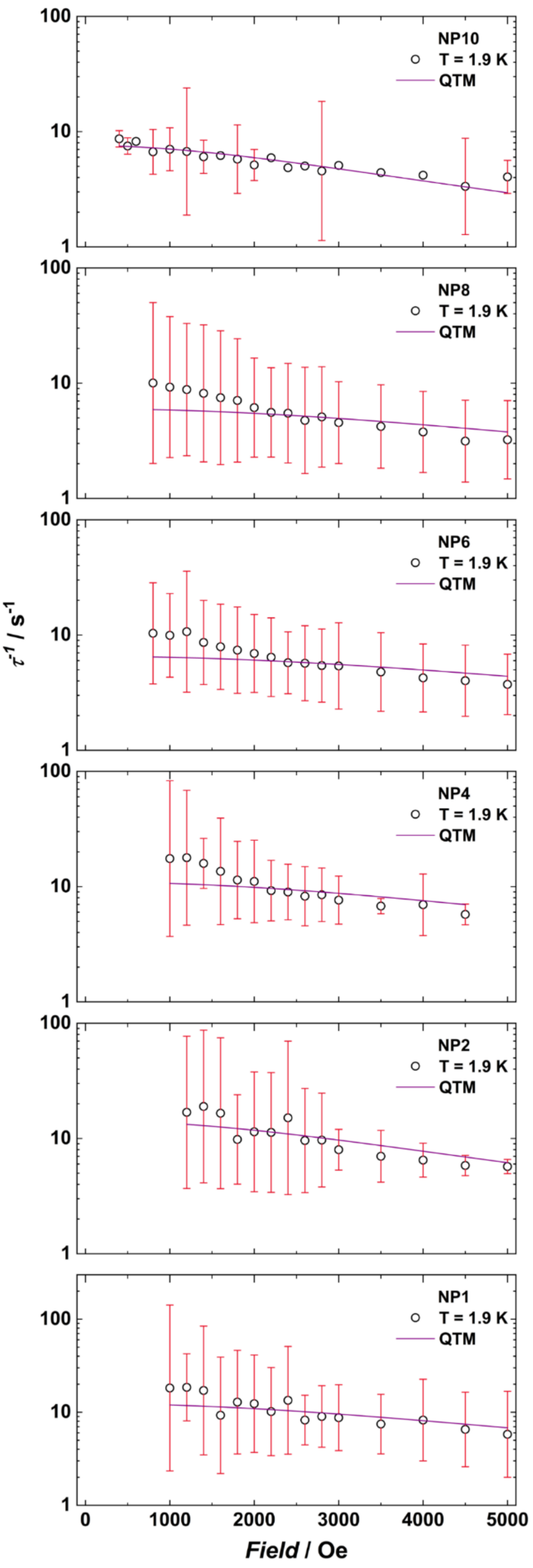

Supplementary Figure 5: **Relaxation mechanism fits.** Field dependence of the magnetization relaxation times (τ) for process 2. For NP10 we were not able to obtain the error for all the points due to the noisy data.

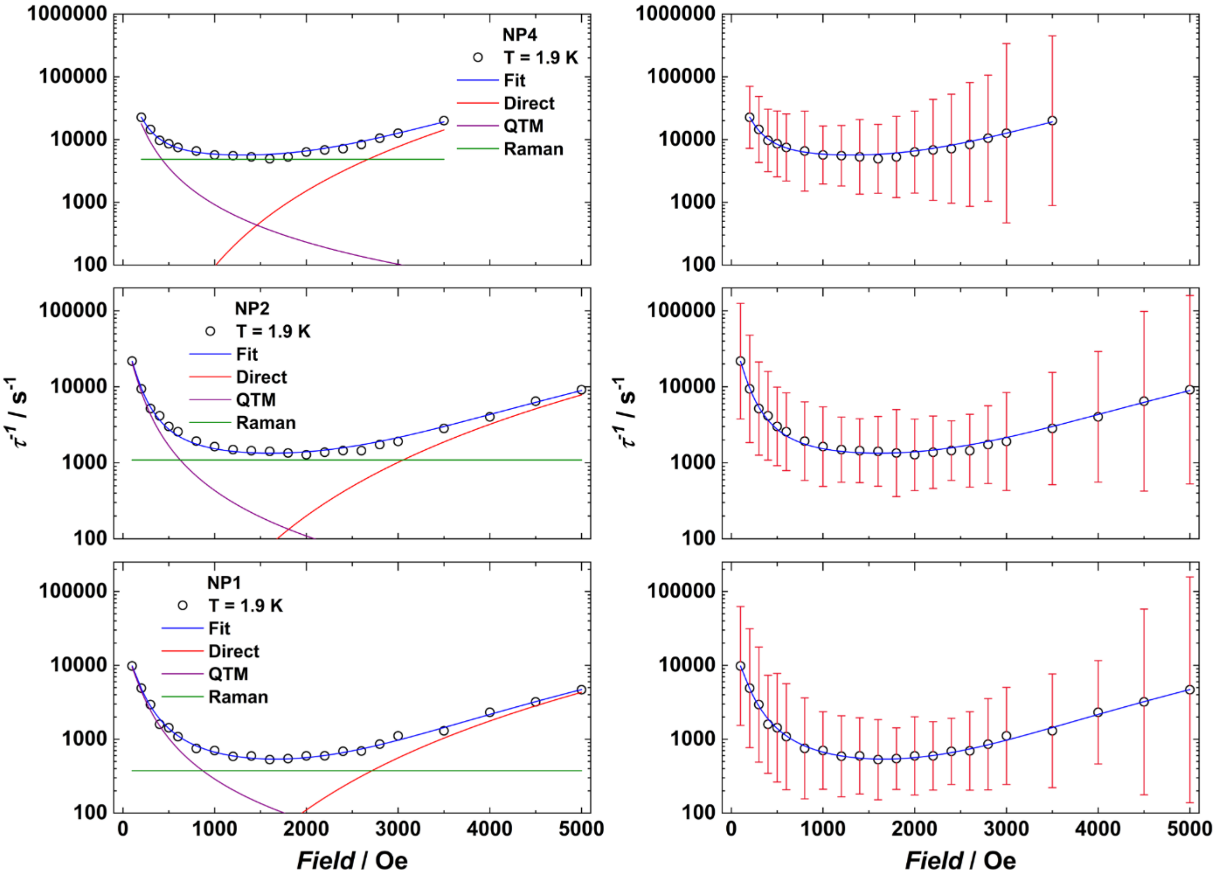

Supplementary Figure 6: **Relaxation mechanism fits.** Field dependence of the magnetization relaxation times (τ) for process 1. Data on left shows the individual contribution for each mechanism. Data on right shows the fitted curve and the ESD for each τ^{-1} value.

Supplementary Table 10: Summary of fit parameters of the field-dependence of the magnetization relaxation times (τ) for the two peaks located in the high (Process 1) and low (Process 2) frequencies.

Nominal Er^{3+} content			Process 1 (high frequency)	Process 2 (low frequency)
NP1	Direct	A	$3.37(0.11) \times 10^{-12}$ $s^{-1}Oe^{-4}K^{-1}$	-
	QTM	B_1	$15.52(0.08) \times 10^{3}$ s^{-1}	$12.35(4.15)$ s^{-1}
		B_2	$6.76(0.66) \times 10^{-5}$ T^{-2}	$3.26(1.42) \times 10^{-8}$ T^{-2}
	Raman	C	$91.40(2.73)$ s^{-1} K^{-n}	-
		n	2.66(0.12)	
NP2	Direct	A	$5.98(0.29) \times 10^{-12}$ $s^{-1}Oe^{-4}K^{-1}$	-
	QTM	B_1	$44.43(0.47) \times 10^{3}$ s^{-1}	$14.26(3.78)$ s^{-1}
		B_2	$1.20(0.21) \times 10^{-4}$ T^{-2}	$5.25(1.46) \times 10^{-8}$ T^{-2}
	Raman	C	$96.62(3.94)$ s^{-1} K^{-n}	-
		n	4.03(0.22)	
NP4	Direct	A	$4.99(0.16) \times 10^{-11}$ $s^{-1}Oe^{-4}K^{-1}$	-
	QTM	B_1	$77.50(17.2) \times 10^{3}$ s^{-1}	$10.96(4.32)$ s^{-1}
		B_2	$8.34(3.62) \times 10^{-5}$ T^{-2}	$2.82(1.76) \times 10^{-8}$ T^{-2}
	Raman	C	$49.51(2.05)$ s^{-1} K^{-n}	-
		n	7.14(0.43)	
NP6	Direct	A	Outside of the experimental range	-
	QTM	B_1		$6.54(2.02)$ s^{-1}
		B_2		$1.97(1.12) \times 10^{-8}$ T^{-2}
	Raman	C		-
		n		
NP8	Direct	A	Outside of the experimental range	-
	QTM	B_1		$5.99(2.31)$ s^{-1}
		B_2		$2.35(1.43) \times 10^{-8}$ T^{-2}
	Raman	C		-
		n		
NP10	Direct	A	Outside of the experimental range	-
	QTM	B_1		$5.08(1.98)$ s^{-1}
		B_2		$8.27(3.75) \times 10^{-9}$ T^{-2}
	Raman	C		-
		n		

Supplementary Table 11: Best-fit parameters to the generalized and double Debye model for the temperature dependence of the the out-of-phase (χ'') magnetic susceptibility for **NP10**. Data collected with an applied field of 1800 Oe (Fig. 3).

T / K	χ_{s1}	χ_{t1}	α_1	τ_1	χ_{s2}	χ_{t2}	α_2	τ_2
2.6	-	-	-	-	0.73997	0.74003	0.061	2.19×10^{-1}
2.4	-	-	-	-	0.73996	0.74004	0.109	2.03×10^{-1}
2.2	-	-	-	-	0.73994	0.74006	0.034	2.01×10^{-1}
2	-	-	-	-	0.73991	0.74009	0.022	2.01×10^{-1}
1.8	-	-	-	-	0.7399	0.7401	0.017	1.79×10^{-1}

Supplementary Table 12: Best-fit parameters to the generalized and double Debye model for the temperature dependence of the the out-of-phase (χ'') magnetic susceptibility for **NP8**. Data collected with an applied field of 1800 Oe (Fig. 3).

T / K	χ_{s1}	χ_{t1}	α_1	τ_1	χ_{s2}	χ_{t2}	α_2	τ_2
2.8	-	-	-	-	0.73997	0.74003	0.261	1.68×10^{-1}
2.6	-	-	-	-	0.73997	0.74003	0.135	1.54×10^{-1}
2.4	-	-	-	-	0.73995	0.74005	0.219	1.49×10^{-1}
2.2	-	-	-	-	0.73994	0.74006	0.170	1.63×10^{-1}
2	-	-	-	-	0.73993	0.74007	0.169	1.88×10^{-1}
1.8	-	-	-	-	0.73992	0.74008	0.095	1.90×10^{-1}

Supplementary Table 13: Best-fit parameters to the generalized and double Debye model for the temperature dependence of the the out-of-phase (χ'') magnetic susceptibility for **NP6**. Data collected with an applied field of 1800 Oe (Fig. 3).

T / K	χ_{s1}	χ_{t1}	α_1	τ_1	χ_{s2}	χ_{t2}	α_2	τ_2
2.8	-	-	-	-	0.73999	0.74001	0.085	1.73×10^{-1}
2.6	-	-	-	-	0.73997	0.74003	0.089	1.55×10^{-1}
2.4	-	-	-	-	0.73997	0.74003	0.084	1.41×10^{-1}
2.2	-	-	-	-	0.73995	0.74005	0.076	1.51×10^{-1}
2	-	-	-	-	0.73993	0.74007	0.131	1.47×10^{-1}
1.8	-	-	-	-	0.73991	0.74009	0.142	1.55×10^{-1}

Supplementary Table 14: Best-fit parameters to the generalized and double Debye model for the temperature dependence of the the out-of-phase (χ'') magnetic susceptibility for **NP4**. Data collected with an applied field of 1800 Oe (Fig. 3).

T / K	χ_{s1}	χ_{t1}	α_1	τ_1	χ_{s2}	χ_{t2}	α_2	τ_2
2.6	0.74471	0.74529	0.445	2.09×10^{-5}	0.73999	0.74001	0.145	1.17×10^{-1}
2.4	0.74474	0.74526	0.405	4.27×10^{-5}	0.73998	0.74002	0.134	8.14×10^{-2}
2.2	0.74478	0.74522	0.333	1.04×10^{-4}	0.73997	0.74003	0.183	8.43×10^{-2}
2	0.74477	0.74523	0.353	1.41×10^{-4}	0.73996	0.74004	0.166	9.10×10^{-2}
1.8	0.7448	0.7452	0.279	2.53×10^{-4}	0.73994	0.74006	0.167	8.79×10^{-2}

Supplementary Table 15: Best-fit parameters to the generalized and double Debye model for the temperature dependence of the the out-of-phase (χ'') magnetic susceptibility for **NP2**. Data collected with an applied field of 1800 Oe (Fig. 3).

T / K	χ_{s1}	χ_{t1}	α_1	τ_1	χ_{s2}	χ_{t2}	α_2	τ_2
3.4	0.74997	0.75011	0.453	7.68×10^{-5}	-	-	-	-
3.2	0.74996	0.75012	0.464	8.54×10^{-5}	-	-	-	-
3	0.74995	0.75014	0.493	8.48×10^{-5}	-	-	-	-
2.8	0.74994	0.75014	0.483	1.06×10^{-4}	-	-	-	-
2.6	0.74495	0.74505	0.144	2.31×10^{-4}	0.73996	0.74004	0.548	1.60×10^{-3}
2.4	0.74494	0.74506	0.209	3.26×10^{-4}	0.73997	0.74003	0.506	8.77×10^{-3}
2.2	0.74491	0.74509	0.258	4.97×10^{-4}	0.73998	0.74001	0.325	6.32×10^{-2}
2	0.74491	0.74509	0.240	6.10×10^{-4}	0.73998	0.74002	0.328	6.90×10^{-2}
1.8	0.74491	0.74509	0.202	8.83×10^{-4}	0.73998	0.74002	0.274	8.08×10^{-2}

Supplementary Table 16: Best-fit parameters to the generalized and double Debye model for the temperature dependence of the the out-of-phase (χ'') magnetic susceptibility for **NP1**. Data collected with an applied field of 1800 Oe (Fig. 3).

T / K	χ_{s1}	χ_{t1}	α_1	τ_1	χ_{s2}	χ_{t2}	α_2	τ_2
3.6	0.75002	0.75007	0.425	2.36×10^{-4}	-	-	-	-
3.4	0.75001	0.75007	0.458	3.01×10^{-4}	-	-	-	-
3.2	0.75001	0.75007	0.383	3.96×10^{-4}	-	-	-	-
3	0.75001	0.75007	0.384	5.63×10^{-4}	-	-	-	-
2.8	0.74496	0.74502	0.332	6.74×10^{-4}	-	-	-	-
2.6	0.74497	0.74503	0.300	8.36×10^{-4}	0.74	0.74	0.377	7.47×10^{-2}
2.4	0.74497	0.74503	0.263	1.03×10^{-3}	0.73999	0.74001	0.259	6.89×10^{-2}
2.2	0.74496	0.74504	0.265	1.33×10^{-3}	0.73999	0.74001	0.262	8.03×10^{-2}
2	0.74496	0.74504	0.261	1.62×10^{-3}	0.73999	0.74001	0.244	9.31×10^{-2}
1.8	0.74496	0.74504	0.207	2.09×10^{-3}	0.73999	0.74001	0.278	9.36×10^{-2}

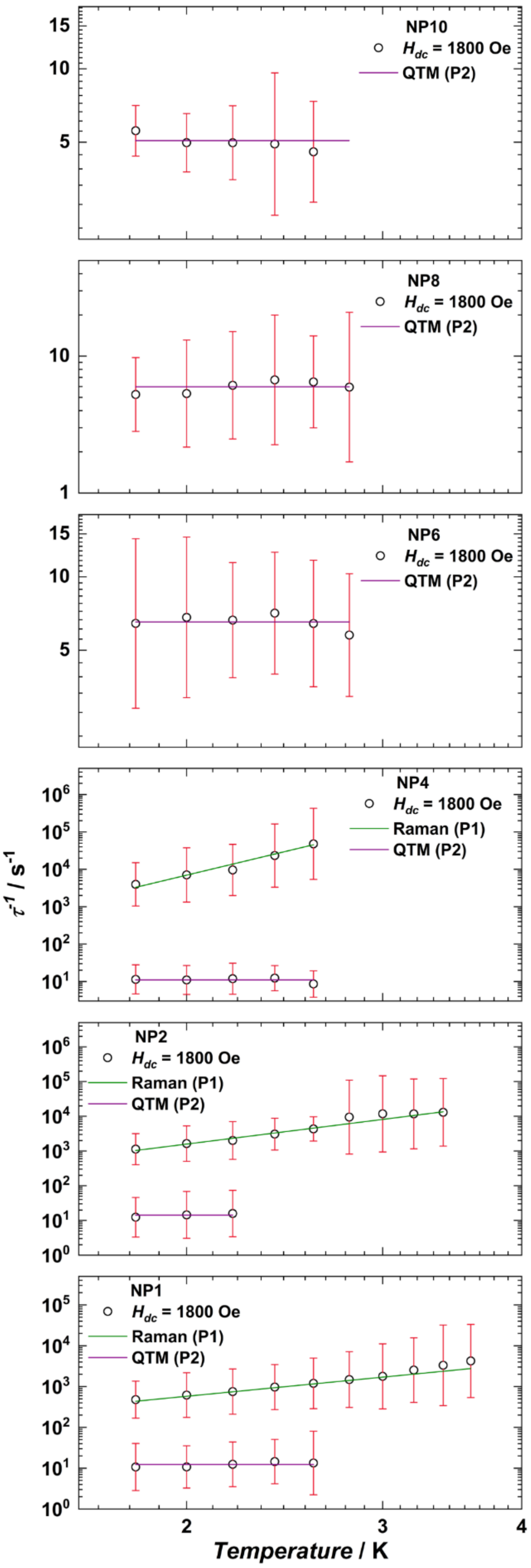

Supplementary Figure 7: **Relaxation mechanism fits.** Log-log plots for the temperature dependence of the magnetization relaxation times (τ) for process 1 (P1) and process 2 (P2).

Supplementary Table 17: Summary of fit parameters of the temperature-dependence of the magnetization relaxation times (τ) for the two peaks located in the high (Process 1) and low (Process 2) frequencies.

Sample			Process 1 (high frequency)	Process 2 (low frequency)
**NP1**	**QTM**	τ_{qtm}	-	8.10(0.49) x 10^{-2} s
	**Raman**	C	91.40(1.16) s^{-1} K^{-n}	-
		n	2.66(0.12)	
**NP2**	**QTM**	τ_{qtm}	-	7.01(0.50) x 10^{-2} s
	**Raman**	C	96.62(16.92) s^{-1} K^{-n}	-
		n	4.03(0.15)	
**NP4**	**QTM**	τ_{qtm}	-	9.12(0.53) x 10^{-2} s
	**Raman**	C	49.51(2.45) s^{-1} K^{-n}	-
		n	7.14(0.22)	
**NP6**	**QTM**	τ_{qtm}	Outside of the experimental range	1.53(0.04) x 10^{-1} s
	**Raman**	C		-
		n		
**NP8**	**QTM**	τ_{qtm}	Outside of the experimental range	1.67(0.07) x 10^{-1} s
	**Raman**	C		-
		n		
**NP10**	**QTM**	τ_{qtm}	Outside of the experimental range	1.97(0.06) x 10^{-1} s
	**Raman**	C		-
		n		

5. Computational Results

To simulate the vibrational normal modes, spin phonon dispersion relations, phonon density of states (DOS), and projected DOS for α-$NaYF_4$ and α-$NaErF_4$, periodic boundary DFT and phonon calculations were carried out for α-$NaYF_4$ using the CRYSTAL17[1] and Phonopy[2, 3] program packages, respectively. The effect of Er^{3+} ions on the aforementioned properties, were taken into account after the geometry optimization of α-$NaYF_4$ in the Γ-point vibrational frequency calculations (see below). Because the CRYSTAL17 cannot treat the half occupancy of Na^+ and Y^{3+} ions on the octahedral site of cubic-phase α-$NaYF_4$, the planar-like model system consisting of alternating layers of Na^+ and Y^{3+} ions was utilized in the solid-state calculations (Figure S8 and S9). This reduce the lattice symmetry of the model system to the tetragonal one, namely P4/mmm (D_{4h}), that is a subgroup of Fm$\overline{3}$m (O_h). Similar approximation has been utilized before in the periodic boundary calculations for the cubic-phase α-$NaYF_4$.[4] Despite the lower symmetry of the model system, the lattice parameters and atomic positions of the tetragonal model system were described well compared to the experimental data (Table S18). For example, the average bond length of the calculated Na–F and Y–F bonds is 2.363 Å, which is in a good agreement with the experimental value of 2.369 Å. The F···F distances are slightly underestimated in the tetragonal model system, but this has only minor effect on its lattice parameters and cell volume. The volume of the optimized cell is only 1 % of smaller than the conventional cell of cubic-phase α-$NaYF_4$ if the lattice parameters of the tetragonal model system is converted back to the cubic one and compared to the original one. The visual inspection of large supercells of the cubic- and tetragonal-phase also illustrates that the tetragonal model system reflects well the lattice structure of the cubic-phase (Figure S9).

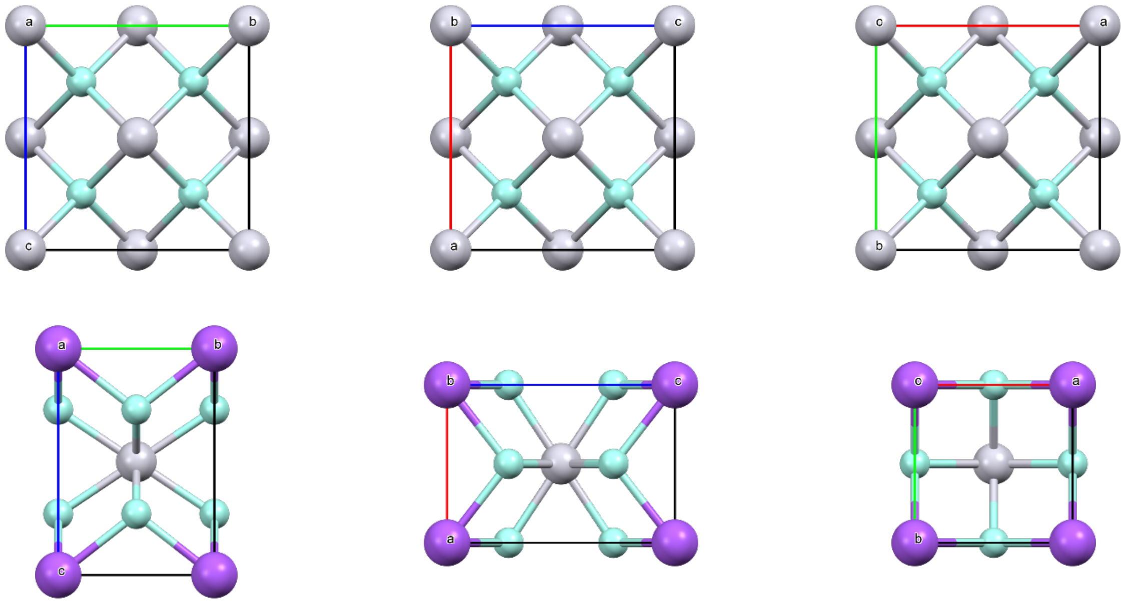

Supplementary Figure 8: Unit cell model for solid-state calculations. *Top row*: Conventional cell (1 × 1 × 1) of cubic-phase α-$NaYF_4$ along a- (left), b- (middle), and c-axis (right). *Bottom row*: Conventional cell (1 × 1 × 1) of tetragonal model system α-$NaYF_4$ utilized in the solid-state calculations along a- (left), b- (middle), and c-axis (right). Color code for the cubic cell: grey = Y^{3+}/Na^+ and pale turquoise = F^-. Color code for the tetragonal cell: grey = Y^{3+}, purple = Na^+, and pale turquoise = F^-. The lattice parameters and distances between atoms are given in Table S18.

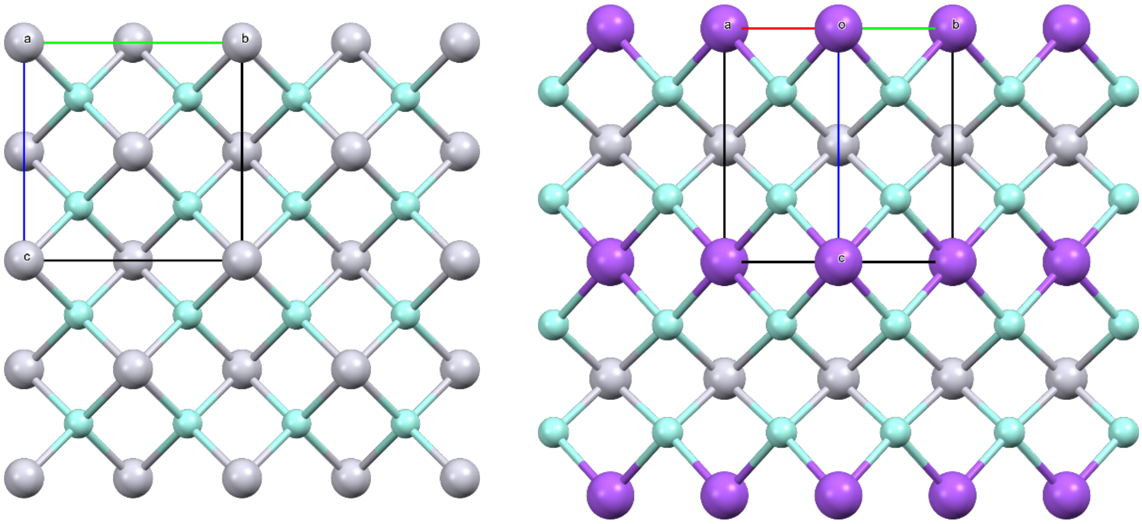

Supplementary Figure 9: Unit cell model for solid-state calculations. 2.0 × 2.0 × 2.0 supercell of cubic α-$NaYF_4$ (left) and 2.5 × 2.5 × 2.0 supercell of tetragonal model system α-$NaYF_4$ utilized in the periodic boundary calculations (right). Color code for the cubic cell: grey = Y^{3+}/Na^+ and pale turquoise = F^-. Color code for the tetragonal cell: grey = Y^{3+}, purple = Na^+, and pale turquoise = F^-. The larger supercells illustrate that the planar-like tetragonal model system reflect the lattice structure of the cubic-phase well.

Supplementary Table 18: Experimental and calculated lattice parameters for the cubic and tetragonal cells, respectively, as well as the experimental and calculated distances between F···F, Na–F, and Y–F atoms. Tetragonal to cubic shows the lattice parameters for the tetragonal cell if it is converted back to the cubic one.

**Compound**	***a* / Å**	***b* / Å**	***c* / Å**	***α* / °**	***β* / °**	***γ* / °**	***V* / Å^3**
Cubic (exp)	5.470	5.470	5.470	90.000	90.000	90.000	163.667
Tetragonal (calc)	3.826	3.826	5.537	90.000	90.000	90.000	81.051
Tetragonal to cubic (calc)	5.411	5.411	5.537	90.000	90.000	90.000	162.115
**Distances**	**F···F / Å**			**Na–F / Å**		**Y–F / Å**	
Cubic (exp)	2.735			2.369		2.369	
Tetragonal (calc)	2.550 & 2.706			2.427		2.299	

In all periodic boundary DFT calculations, the Perdew–Burke-Ernzerhof (PBE) GGA functional[5] in conjunction with pob-TZVP triple-zeta valence[6, 7] basis sets designed for the solid-state calculations were employed. For the evaluation of the Coulomb and exchange integrals tight tolerance factors (TOLINTEG) of 8, 8, 8, 8, and 16 were used. The SCF convergence criteria (TOLDEE) were set to 10^{-7} and 10^{-10} in optimization and Γ-point vibrational frequency calculations, respectively. The very tight SCF convergence criteria (10^{-10}) were also used in the second order force constant calculations of the displaced structures (see below). k-points within the Brillouin zone of the reciprocal space were sampled with the Monkhorst-pack method applying a grid of 12 × 12 × 12 in the atomic positions and unit cell optimizations as well as in the Γ-point vibrational frequency calculations, whereas the grids of 6 × 6 × 6 and 3 × 3 × 4 were used in the second order force constant calculations carried out for the 2 × 2 × 2 and 4 × 4 × 3 supercells of tetragonal model system, respectively. Otherwise, default parameters of CRYSTAL17 were utilized in the optimizations, Γ-point vibrational frequency, and second order force constant calculations. The visualization of the vibration modes was done with the CRYSPLOT.[8] The output files needed for the visualization of vibration modes are provided in the ESI.

The dispersion relations, DOS, and projected DOS were modelled with the Phonopy program without and with a non-analytical term correction.[9] Tetrahedron method was used in the DOS and projected DOS calculations. The mesh sizes of 101 × 101 × 101, 101 × 101 × 101, 41 × 41 × 41, were utilized in the mesh sampling of dispersion relations,

DOS, and projected DOS, respectively. All other parameters were kept as defaults. Phonon calculations were performed for the 2 × 2 × 2 and 4 × 4 × 3 supercells.

The following procedure was used to obtain the vibrational modes, phonon dispersion relations, DOS, and projected DOS for α-$NaYF_4$ and α-$NaErF_4$. First, the atomic positions and unit cell parameters of α-$NaYF_4$ were optimized in the space group of P4/mmm. It should be noted that the structure of α-$NaYF_4$ was also optimized without symmetry constraint in the space group of P1, but this led to the zig-zag arrangement of F^- anions and stronger deviation from the cubic symmetry. Also, the geometry optimization for the 2 × 2 × 2 supercells of α-$NaYF_4$ retaining the cubic symmetry were unsuccessful and led to the solutions that were not minimum on the potential energy hypersurface or not converged in the set convergence criteria. Thus, the tetragonal model system α-$NaYF_4$ was utilized in the subsequent Γ-point vibrational frequency calculations. Second, two different Γ-point vibrational frequency calculations were carried out for the optimized lattice of α-$NaYF_4$. One where the standard isotope mass of Na, Y and F were used and other where the mass of Y^{3+} ion was replaced with standard atomic mass of Er^{3+} ion (167.259). The latter simulates the effect of Er^{3+} ions on the vibration spectrum, phonon dispersion relations, DOS, and project DOS of α-$NaYF_4$. Given the similar ionic radii of Y^{3+} and Er^{3+} and the fact that Y and Er compounds are usually isostructural, the above approximation is valid and is routinely utilized in *ab initio* and DFT calculations. Importantly, by using this approximation there is no need to optimize the atomic positions and unit cell parameters of α-$NaErF_4$ that can easily lead to the convergence problems in the SCF steps due to the open shell nature of Er^{3+} ions. Third, after the Γ-point vibrational frequency calculations for α-$NaYF_4$ and α-$NaY^{167.259}F_4$, the displaced structures for their 2 × 2 × 2 and 4 × 4 × 3 supercells were generated from the output files of the Γ-point vibrational frequency calculations using the Phonopy program. Fourth, the second order force constants for all displaced structures were calculated with the CRYSTAL17 program after which the supercell outputs were processed with the Phonon program to obtain phonon dispersion relations, DOS, and projected DOS without and with a non-analytical term correction for α-$NaYF_4$ and α-$NaY^{167.259}F_4$. Figure S10 reports the calculated vibrational modes for the tetragonal α-$NaYF_4$ and α-$NaY^{167.259}F_4$ and their calculated phonon dispersion relations, DOS, and projected DOS are given in Figures S11–S13. Similar results obtained for the 2 × 2 × 2 and 4 × 4 × 3 supercells indicate that the latter supercells are large enough to obtain converged results in phonon calculations.

Thus, only the results obtained for the 4 × 4 × 3 supercells are discussed in more detail. Any attempt to increase the size of the supercells above 4 × 4 × 3 led to significant increase in the computational time.

The vibration spectrum of α-$NaYF_4$ is a rather sparse and many of the vibration modes are degenerated due to the high symmetry of α-$NaYF_4$ (Figure S10 and Table S19). The most intense Raman active bands at 342 cm^{-1}, 349 cm^{-1} (masked by the band at 342 cm^{-1}) and 413 cm^{-1} are in good agreement with the previously reported values of 329 cm^{-1} and 419 cm^{-1}.[10] These are characteristic Raman active vibration modes for the cubic-phase α-$NaYF_4$ and their visualization reveals that they are associated to the displacements of F atoms around the coordination sphere of Y atoms, which is further supported by the calculated projected DOS (Figure 12). F atoms dominates the phonon distribution above ~250 cm^{-1} (Figure S12). The least intense Raman band at 169 cm^{-1} shows the wagging of YF_8 unit, but no contribution from Na atoms although in this region of spectrum Na atoms show small but non-negligible contribution on the phonon distribution (Figure S12). The first four intense IR bands at 182 cm^{-1}, 229 cm^{-1} (a shoulder-type peak), 239 cm^{-1} and 318 cm^{-1} are characterized by the simultaneous movement of Na and Y atoms with some contribution from F atoms. Out of these four vibrations bands, the shoulder-type peak at 229 cm^{-1} contains the most intense contribution from Na atoms that could be expected on the base of the calculated projected DOS (Figure S12). The fifth intense IR active vibration band at 334 cm^{-1} is again mainly associated to the movement of F atoms. Similar analysis can also be done for the vibration modes of α-$NaY^{167.259}F_4$. However, they are clearly shifted if compared to the vibration modes of α-$NaYF_4$ because heavier Er ($Y^{167.259}$) atoms, shift the calculated vibration modes towards lower wavenumbers as expected. This effect is more pronounced for the IR active vibration modes than for the Raman active modes. Because the studied Er doped NPs consist of both Y and Er ions, it is expected that their lattice vibrations resemble the ones obtained for both, α-$NaYF_4$ and α-$NaY^{167.259}F_4$. The results suggest that the positions of the lattice vibrations in NPs based on α-$NaYF_4$ can be altered by the nature of doped atoms. Indeed, the doped NPs based on cubic- or hexagonal-phase of $NaYF_4$ incorporating different paramagnetic lanthanide ions show distinct but very characteristic vibrational spectra that widths can vary from ~ 500 cm^{-1} to ~1400 cm^{-1}.[11-21] This provides the direct means to tune the positions and amounts of the lattice vibrations in doped $NaYF_4$ NPs. The positions and amounts of the lattice vibrations are important factors in

the design of materials that show slow relaxation of magnetization because vibrations couple with the spins of paramagnetic lanthanide centers via the spin-phonon coupling and mediate magnetic relaxation if the energies of electronic and vibration states are in resonance and the nature of vibration is correct.[22] The latter implies that the coordination sphere of paramagnetic lanthanide ion should be involved in the vibration to induce a (strong) spin-phonon coupling (see discussion below). For example, in the studied NPs the optical phonon modes with high DOS at the region of 300–350 cm^{-1} are associated to the movement of F atoms around the first coordination sphere of Er atoms and coincide with the calculated energies of the four highest excited KDs – degenerated pairs of **KD5** & **KD6** and **KD7** & **KD8** – of the fragment **$[Na_8Y_4ErF_{24}]^-$** (see below, Figure S13, Table S20 and S24). Thus, these vibration modes could facilitate spin-phonon coupling and magnetic relaxation in the studied Er doped NPs. Otherwise, the energies of the calculated KDs of the fragment **$[Na_8Y_4ErF_{24}]^-$** are in an energy range where the phonon DOS is low and phonon modes originate from acoustic phonons.

The first optical phonon mode of α-$NaYF_4$ is observed at 125 cm^{-1} below which the DOS is dictated by acoustic phonons, whereas in the region of 125–177 cm^{-1} both, acoustic and optical, phonons contribute to the DOS, and above 177 cm^{-1} optical phonons dominate the DOS (Figure S12). Even though no clear phonon gap is observed for α-$NaYF_4$, it has two different regions, ~170–260 cm^{-1} and ~295–360 cm^{-1}, where phonons are accumulated, otherwise the phonon DOS is low compared to high density regions. Comparable results have also been found in the previous studies for α-$NaYF_4$.[23, 24] Similar analysis can also be done for α-$NaY^{167.259}F_4$ from Figure S13. Interestingly, the large part of phonon distributions originating from Er ($Y^{167.259}$) atoms is located at the low energy part of the phonon spectrum where only acoustic phonons are present. Because phonons that are associated to paramagnetic centers usually give the large spin-phonon couplings, it is highly likely that acoustic phonons play a more important role in the magnetic relaxation processes of the studied Er doped NPs than optical phonons. This is further support by the fact that the thermal population of optical phonons should be rather low in the temperature region (< 3.8 K) where the Er doped NPs show the magnetic relaxation – the first optical phonon mode is observed at 122 cm^{-1} for α-$NaY^{167.259}F_4$. However, the role of optical phonons on the magnetic relaxation processes of the studied NPs cannot fully be ruled out by this simplified analysis because the vibrations of atoms on the first and second coordination sphere of paramagnetic center can also lead to the

strong spin-phonon couplings. Indeed, F atoms, that are on the first coordination sphere of Er^{3+} ion, do not only have phonon distribution on the acoustic region, but also show high phonon distribution on the optical bands.

Whilst the above qualitative analysis does not indicate how strongly or weakly each lattice vibrations of α-$NaYF_4$ and α-$NaY^{167.259}F_4$ are coupled to the spin of Er^{3+} ions as these are only obtained from the spin-phonon coupling calculations, the other obtained results can be compared to molecular compounds which lattice vibrations usually span from 0 cm^{-1} to 3500 cm^{-1} and phonon spectra typically show several regions where the phonon DOS is high.[22, 25, 26] Thus, if the paramagnetic lanthanide ions should be incorporated into the host matrix that provide a small amount of vibrational modes and sparse phonon spectrum, NPs based on α-$NaYF_4$ or other inorganic solids offer a competitive alternative to molecular compounds. Moreover, the position of vibration bands and phonon spectrum of $NaYF_4$ based NPs can be tuned in rational manner by changing the nature and concentration of doped atom from one to other as exemplified by the previously reported data.[11-21]

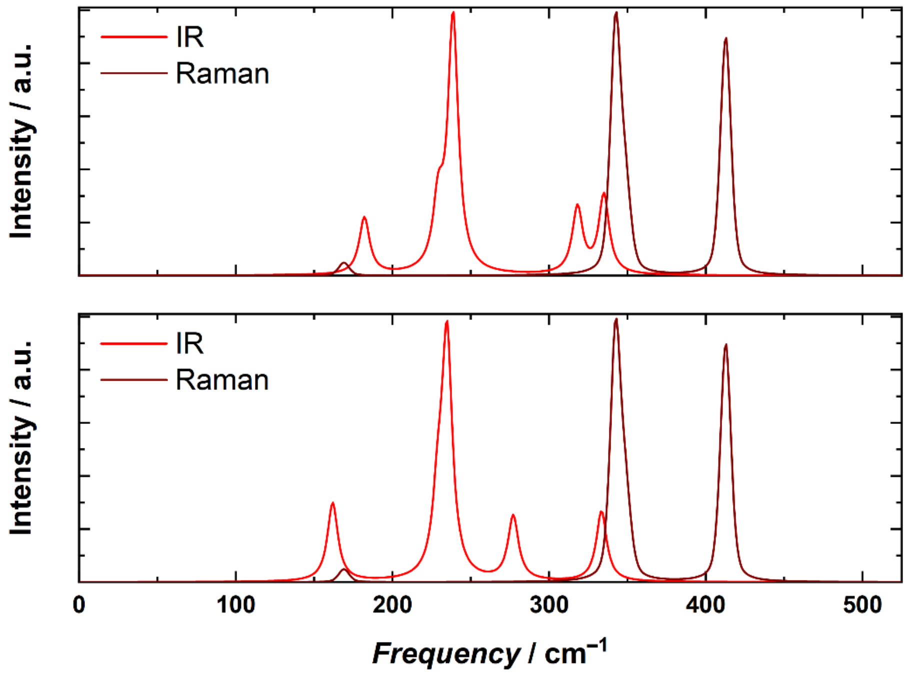

Supplementary Figure 10: Calculated IR and Raman spectra for the tetragonal model systems α-$NaYF_4$ (top) and α-$NaY^{167.259}F_4$ (bottom). IR and Raman active modes are visualized with red and dark, respectively. All calculated vibrations modes along with their intensities for α-$NaYF_4$ and α-$NaY^{167.259}F_4$ are also tabulated in Table S19 and S20, respectively.

Supplementary Table 19: Calculated vibration modes for the tetragonal model system α-$NaYF_4$. IR and Raman intensities are given in arbitrary units. The vibrational mode with the highest intensity is normalized to one and intensities of all other modes are scaled with respect to the mode with the highest intensity.

Frequency	Irrep	IR active	Intensity	Raman active	Intensity
0	A_{2u}	Yes	0.000	-	-
0	E_u	Yes	0.000	-	-
165	B_{2u}	-	-	-	-
170	E_g	-	-	Yes	0.052
182	E_u	Yes	0.222	-	-
229	A_{2u}	Yes	0.252	-	-
239	E_u	Yes	1.000	-	-
318	A_{2u}	Yes	0.260	-	-
335	E_u	Yes	0.309	-	-
342	E_g	-	-	Yes	1.000
349	B_{1g}	-	-	Yes	0.291
413	A_{1g}	-	-	Yes	0.958

Supplementary Table 20: Calculated vibration modes for the tetragonal model system $NaY^{167.259}F_4$. IR and Raman intensities are given in arbitrary units. The vibrational mode with the highest intensity is normalized to one and intensities of all other modes are scaled with respect to the mode with the highest intensity.

Frequency	Irrep	IR active	Intensity	Raman active	Intensity
0	E_u	Yes	0.000	-	-
0	A_{2u}	Yes	0.000	-	-
162	E_u	Yes	0.328	-	-
165	B_{2u}	-	-	-	-
170	E_g	-	-	Yes	0.052
229	A_{2u}	Yes	0.292	-	-
235	E_u	Yes	1.000	-	-
277	A_{2u}	Yes	0.270	-	-
333	E_u	Yes	0.294	-	-
342	E_g	-	-	Yes	1.000
349	B_{1g}	-	-	Yes	0.166
413	A_{1g}	-	-	Yes	0.888

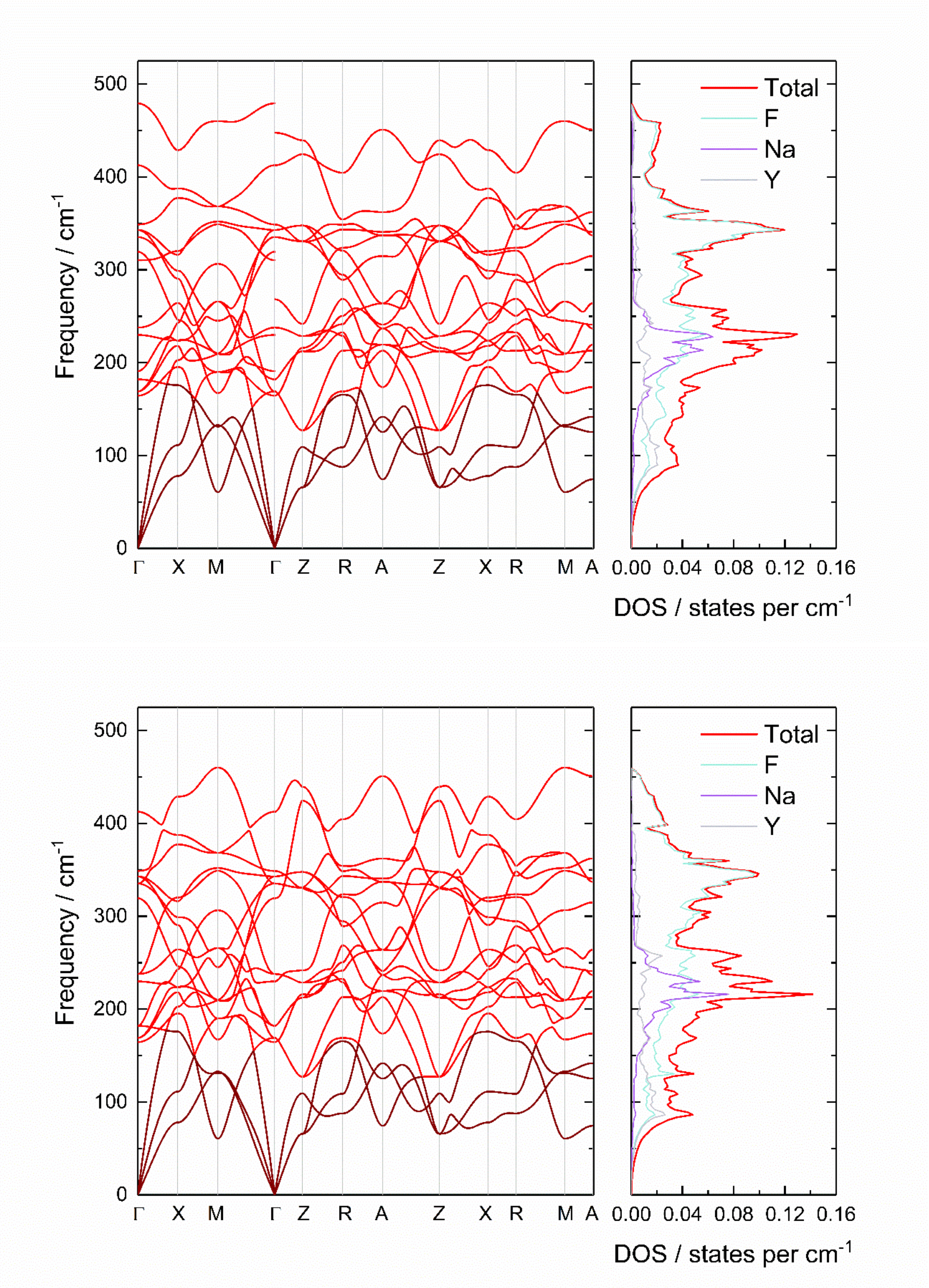

Supplementary Figure 11: Calculated phonon dispersion relations, DOS, and projected DOS for the 2 × 2 × 2 supercell of tetragonal model system α-$NaYF_4$ without (bottom) and with a non-analytical term correction (top). Optical and acoustic phonon bands are described with red and dark, respectively. The discontinuation of optical phonon bands at Γ points arises from LO-TO splitting which is taken into account by the non-analytical term correction.

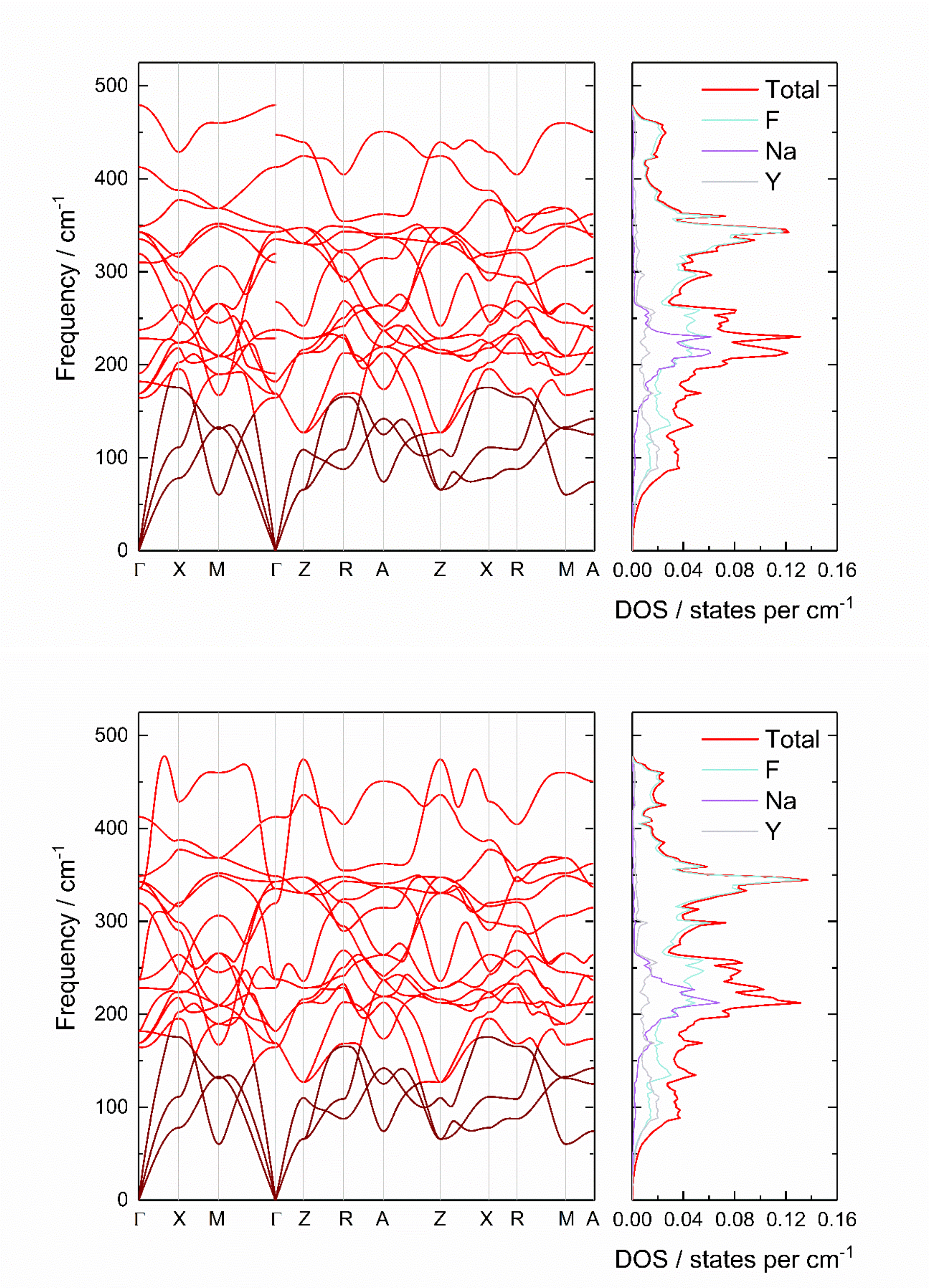

Supplementary Figure 12: Calculated phonon dispersion relations, DOS, and projected DOS for the 4 × 4 × 3 supercell of tetragonal model system α-$NaYF_4$ without (bottom) and with a non-analytical term correction (top). Optical and acoustic phonon bands are described with red and dark, respectively. The discontinuation of optical phonon bands at Γ points arises from LO-TO splitting which is taken into account by the non-analytical term correction.

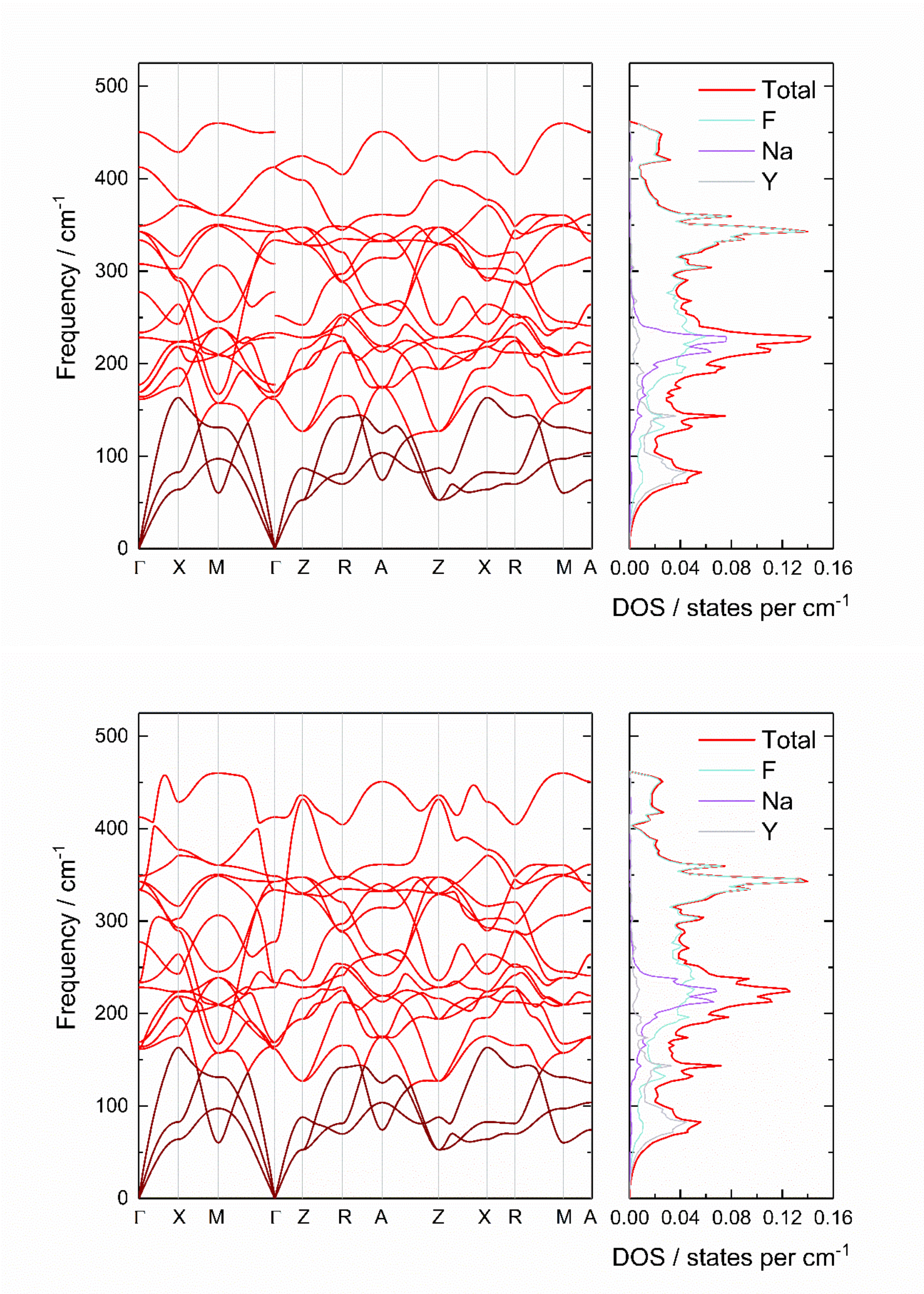

Supplementary Figure 13: Calculated phonon dispersion relations, DOS, and projected DOS for the 4 × 4 × 3 supercell of tetragonal model system α-$NaY^{167.259}F_4$ without (bottom) and with a non-analytical term correction (top). Optical and acoustic phonon bands are described with red and dark, respectively. The discontinuation of optical phonon bands at Γ points arises from LO-TO splitting which is taken into account by the non-analytical term correction.

Ab initio calculations were performed for six different NP fragments – $\mathbf{[Na_8Y_4ErF_{22}]^+}$, $\mathbf{[Na_8Y_4ErF_{23}]}$, $\mathbf{[Na_8Y_4ErF_{24}]^-}$, $\mathbf{[Na_8Y_{10}ErF_{40}]^+}$, $\mathbf{[Na_8Y_{10}ErF_{41}]}$, and $\mathbf{[Na_8Y_{10}ErF_{42}]^-}$ – using OPENMOLCAS 19.11 quantum chemistry software.[27] The fragments were created from the reported crystal structure of $NaYF_4$ by taking various sets of atoms and replacing the single Y^{3+} ion with Er^{3+} in the middle of each model.[28] The ANO-RCC-VTZP basis set was used for the Er^{3+} ion, and the ANO-RCC-VDZP for Y^{3+}, Na^+ and F^- ions.[29, 30] The scalar relativistic effects were treated using the exact two-component (X2C) transformation.[31-33] The Cholesky decomposition was used for the two-electron integrals with a value of 10^{-8} to reduce the disk space. In the restricted active space self-consistent field (RASSCF)[34-36] calculations, all 35 spin quartets and 112 spin doublets, arising from 11 electrons in seven 4f-orbitals, were solved. All solved 35 quartets and 112 doublets were mixed in the subsequent restricted active space state interaction (RASSI)[37] calculation to take into account the spin-orbit effect. Microscopic magnetic properties of the model systems were then calculated using the SINGLE_ANISO program.[38-40] The same initial coordinate system (Er^{3+} ion at the origin) and quantization axis (Zm; determined by default by the SINGLE_ANISO program) were used for all NP fragments in the SINGLE_ANISO calculations. The dipolar coupling between two Er^{3+} ions separated by the distance r_{12}, were calculated by diagonalisation of the Hamiltonian given in Equation 1 as implemented in the POLY_ANISO program:[41, 42]

$$\hat{H} = \mu_{bohr}^2 \frac{\hat{\boldsymbol{\mu}}_1 \cdot \hat{\boldsymbol{\mu}}_2 - 3(\hat{\boldsymbol{\mu}}_1 \cdot \boldsymbol{n}_{12})(\hat{\boldsymbol{\mu}}_2 \cdot \boldsymbol{n}_{12})}{r_{12}^3}. \quad (1)$$

In Equation 1, μ_{bohr}^2 is the square of Bohr magneton, $\hat{\boldsymbol{\mu}}_1$ and $\hat{\boldsymbol{\mu}}_2$ are magnetic moments on the sites 1 and 2, respectively, $\boldsymbol{n}_{12}$ is the unit vector connecting the sites 1 and 2, and r_{12} is the distance between the sites 1 and 2. The two lowest states of the $^4I_{15/2}$ ground multiplet of both Er^{3+} ions were used as an exchange basis in the POLY_ANISO calculations. The calculated energies of exchange states obtained by the diagonalisation of Equation 1 are given in Table S21 for each calculated Er^{3+} ion pair.

Supplementary Table 21: Calculated energies (cm^{-1}) of low-lying exchange states for two Er^{3+} ions separated by the distance r_{12}.

Exchange state	r_{12} = 3.868	r_{12} = 5.470	r_{12} = 8.649	r_{12} = 10.940
**1**	0.000	0.000	0.000	0.000
**2**	0.006	0.006	0.002	0.001
**3**	0.069	0.069	0.018	0.009
**4**	0.536	0.189	0.048	0.024

From the energy differences of exchange states, the dipolar coupling parameter J_{dip} was determined for each interacting pair using the following Hamiltonian:

$$\hat{H}_{dip} = -J_{dip}\hat{S}_1\hat{S}_2, \quad (2)$$

in which $\hat{S}_1$ and $\hat{S}_2$ are the projection operators of the pseudo spins (S=1/2) that describe the local ground KD of Er^{3+} ion on the sites 1 and 2, respectively. It should be noted that the exchange doublets are split and the highest energy difference between the ground and first excited exchange doublets was utilized to extract the dipolar coupling parameter J_{dip}. The described procedure is well established and has been used before to calculate the dipolar coupling parameters between interacting metal centres which exchange doublets are split.[43-45]

The constructed NP fragments should simulate the crystal field (C.F.) experienced by the Er^{3+} ion in NPs reasonably well because Monte Carlo simulations and magnetic measurements showed that, in the diluted samples, Er^{3+} ions are isolated in a diamagnetic matrix and the dipolar interactions between Er^{3+} ions are suppressed. However, we found out that the computational results are sensitive to the size and symmetry of the fragment. Thus, we compared the calculated energies, compositions, and principal (g_x, = g_y = g_z) and average (g_{avg}) g-values of Kramers doublets (KD) of the investigated NP fragments to the recently reported EPR study of $NaY_{0.98}Er_{0.02}F_4$ which confirmed that the ground KD of the $^4I_{15/2}$ ground multiplet of Er^{3+} ion in $NaY_{0.98}Er_{0.02}F_4$ is the Γ_7 doublet state (in Bethe notation).[46] The order of the excited states for $NaY_{0.98}Er_{0.02}F_4$ was concluded to be following, given in increasing order of energy: the $\Gamma_8^{(1)}$ quartet state, Γ_6 doublet state, $\Gamma_8^{(2)}$ quartet state, and $\Gamma_8^{(3)}$ quartet state.[46] The doublet and quartet states consist of one and two Kramers doublets (KD), respectively. It should be noted that in the EPR study of

$NaY_{0.98}Er_{0.02}F_4$ two different energy values were reported for each state of the $^4I_{15/2}$ ground multiplet of Er^{3+} ion because two different Hamiltonians were used in the study:

$$\hat{H}_1 = \mu_{bohr} g_J \vec{H} \cdot \vec{J} + \hat{H}_{C.F.}, \quad (3)$$

$$\hat{H}_2 = \hat{H}_{FI} + \hat{H}_{C.F.}, \quad (4)$$

in which, μ_{bohr}, g_J, $\vec{H}$, $\vec{J}$, and $\hat{H}_{FI}$ are Bohr magneton, Landé factor, external magnetic field, total angular momentum and free electron Hamiltonian, respectively, and $\hat{H}_{C.F.}$ is an approximated C.F Hamiltonian defined in Equation 5 below. The full definition of the free electron Hamiltonian is given in the original paper along with the dc-field magnetization data which was utilized to extract the (C.F.) parameters for $NaY_{0.98}Er_{0.02}F_4$.[46]

The compositions of the eigenfunctions of the experimentally determined states can be derived from the approximate C.F. Hamiltonian, if it assumed that the Er^{3+} ion resides in the perfect cubic C.F. environment in the investigated NPs:

$$\hat{H}_{C.F.} = w\left[x\frac{B_4^0(\hat{O}_4^0+5\hat{O}_4^4)}{F(4)} + (1-|x|)\frac{B_6^0(\hat{O}_6^0-21\hat{O}_6^4)}{F(6)}\right] \quad (5)$$

where w, B_k^q and $\hat{O}_k^q$ are the scale factor, C.F. parameters, and Stevens operator equivalents, respectively, -1 < x < 1, and $F(4)$ and $F(6)$ are the numerical factors as reported by Lee, Leask and Wolf.[47] By diagonalizing this C.F. Hamiltonian the eigenvalues and eigenfunctions of each state can be obtained for a given (ground) state *J*-multiplet. For example, for Er^{3+} ion with J = 15/2, the composition of the wavefunction of the Γ_7 doublet state is $40\%|\pm13/2\rangle + 34\%|\pm5/2\rangle - 20\%|\pm3/2\rangle - 6\%|\pm11/2\rangle$ according to the tabulated values. Similarly, the compositions of the eigenfunctions of other doublet and quartet states can be determined from the tabulated values.

To compare the calculated and experimental *g*-values, the averaged *g*-values were calculated for each KD of the NP fragments using Equation 6:

$$g_{avg} = \frac{g_x+g_y+g_z}{3}. \quad (6)$$

The principal *g*-values (g_x, g_y, g_z) used in Equation 6 were obtained from the *ab initio* calculations of the NP fragments. In the strict sense, Equation 6 only holds for the isotropic (g_x, = g_y = g_z) Γ_7 and Γ_6 doublet states but not for the anisotropic $\Gamma_8^{(1)}$–$\Gamma_8^{(3)}$

quartet states. However, experimental evidence have shown that when the C.F. breaks the symmetry of the cubic environment, the averaged g-values remain close to the isotropic g-values of the Γ_7 and Γ_6 states in the cubic symmetry.[48, 49] By assuming that this also holds for the anisotropic $\Gamma_8^{(1)}$–$\Gamma_8^{(3)}$ quartet states the values of g_{avg} can be utilized in comparison of calculated and experimental data.

An investigation of compositions of the wavefunctions of the fragments shows that the ground KDs of **$[Na_8Y_4ErF_{24}]^-$** and **$[Na_8Y_{10}ErF_{40}]^+$** are composed of the M_J = ±13/2, ± 5/2, ± 3/2, and ± 11/2 states as expected for the system in which the Er^{3+} ion occupies the O_h site symmetry (Table S30 and S31).[47] However, the closer inspection of weights of the M_J components reveals that the weights of **$[Na_8Y_{10}ErF_{40}]^+$** differ from those of **$[Na_8Y_4ErF_{24}]^-$** which are in good agreement with the values obtained from the approximate C.F. Hamiltonian (eq. 5) for the Γ_7 doublet state. Like the ground KD of **$[Na_8Y_4ErF_{24}]^-$**, the ground KDs of **$[Na_8Y_4ErF_{22}]^+$** and **$[Na_8Y_4ErF_{23}]$** are also strongly mixed. The main contribution to their ground KDs still arises from the M_J = ±13/2 states, but a significant contribution originates also from the M_J = ± 15/2 states (Table S28 and S29). The compositions of the ground KDs of **$[Na_8Y_{10}ErF_{41}]$**, and **$[Na_8Y_{10}ErF_{42}]^-$** resemble the one of [**$Na_8Y_{10}ErF_{40}]^+$** with additional mixing from the higher lying states (Table S31–S33). Thus, they cannot be defined as the pure Γ_7 doublet state. These findings suggest that, in the studied fragments, the strong deviation from the O_h symmetry leads to the significant mixing of the Γ_7 doublet state with the excited states, because the point group symmetries of **$[Na_8Y_4ErF_{22}]^+$**, **$[Na_8Y_4ErF_{23}]$**, **$[Na_8Y_4ErF_{24}]^-$**, **$[Na_8Y_{10}ErF_{40}]^+$**, **$[Na_8Y_{10}ErF_{41}]$**, and **$[Na_8Y_{10}ErF_{42}]^-$** are C_i, C_1, D_{4h}, D_{4h}, C_s and C_{2h}, respectively. Two other findings can also be concluded from the data. First, the distortion of the symmetry near the inner coordination sphere of Er^{3+} ion, like in **$[Na_8Y_4ErF_{22}]^+$** and **$[Na_8Y_4ErF_{23}]$**, change the composition of the ground KD more than the distortion far away from the Er^{3+} site, like in **$[Na_8Y_{10}ErF_{41}]$**, and **$[Na_8Y_{10}ErF_{42}]^-$** (Table S28, S29, S32, and S33) Second, defects on the proximity of Er^{3+} ion increase its magnetic anisotropy because, out of all studied fragments, **$[Na_8Y_4ErF_{22}]^+$** and **$[Na_8Y_4ErF_{23}]$** fragments have the most axial ground KDs as revealed by their **g**-tensors (Table S22 and S23).

As explained above, the *ab initio* calculations predicted the strongly mixed ground KDs for **$[Na_8Y_4ErF_{22}]^+$** and **$[Na_8Y_4ErF_{23}]$** that could not be assigned to the pure Γ_7 doublet state, whereas the KDs of **$[Na_8Y_{10}ErF_{41}]$** and **$[Na_8Y_{10}ErF_{42}]^-$** contained small

contributions from the higher lying states but were otherwise similar with the ground KD of **[$Na_8Y_{10}ErF_{40}$]$^+$**. Moreover, the other calculated data – energy spectra, **g**-tensors, and compositions of the excited KDs – for **[$Na_8Y_{10}ErF_{41}$]** and **[$Na_8Y_{10}ErF_{42}$]$^-$** are in line with the results obtained for **[$Na_8Y_{10}ErF_{40}$]$^+$** (Table S25–S27 and S31–S33). Thus, we will only describe the results obtained for the highest symmetry **[$Na_8Y_4ErF_{24}$]$^-$** and **[$Na_8Y_{10}ErF_{40}$]$^+$** fragments in detail.

For **[$Na_8Y_{10}ErF_{40}$]$^+$**, the splitting of the $^4I_{15/2}$ ground multiplet is 354 cm^{-1} and the stabilization of the ground KD is 20 cm^{-1} (Table S25) For **[$Na_8Y_4ErF_{24}$]$^-$**, the same values are 390 cm^{-1} and 40 cm^{-1} (Table S22). These values are in a reasonable agreement with the previously published data for $NaY_{0.98}Er_{0.02}F_4$ for which the splitting of the $^4I_{15/2}$ ground multiplet and ground state stabilization were 309 cm^{-1} (263 cm^{-1}) and 56 cm^{-1} (48 cm^{-1}), respectively, when the values were extracted from the Hamiltonian $\widehat{H}_2$ ($\widehat{H}_1$).[46] Closer inspection of the energy spectrum as well as the weights of M_J components, **g**-tensors and averaged g-values of each KDs of **[$Na_8Y_{10}ErF_{40}$]$^+$** (Table S25 and S31), suggest that the first KD1 and second KD2 are the Γ_6 and Γ_7 doublet state. In particular, the calculated averaged g-values for the KD1 ($g_{avg} = 6.08$) and KD2 ($g_{avg} = 6.59$) indicate that the Γ_6 doublet state is a ground state for **[$Na_8Y_{10}ErF_{40}$]$^+$** instead of the Γ_7 doublet. The experimentally determined g-values for the Γ_6 and Γ_7 doublet states are ~6.0 and 6.8, respectively, in $NaY_{0.98}Er_{0.02}F_4$.[46] The remaining six excited KDs form the three $\Gamma_8^{(1)}$–$\Gamma_8^{(3)}$ quartet states of which the first one, consisting of KD3 and KD4, is clearly split by 43 cm^{-1}. The calculated splitting of the $\Gamma_8^{(1)}$ quartet is larger than predicted from the EPR study of $NaY_{0.98}Er_{0.02}F_4$ (< 28 cm^{-1}). It is evident from the above data, that the *ab initio* calculations fail to produce the correct order of states – Γ_6, Γ_7, $\Gamma_8^{(1)}$, $\Gamma_8^{(2)}$, and $\Gamma_8^{(3)}$ – for **[$Na_8Y_{10}ErF_{40}$]$^+$**. Contrast to **[$Na_8Y_{10}ErF_{40}$]$^+$**, the order of states – Γ_7, Γ_6, $\Gamma_8^{(1)}$, $\Gamma_8^{(2)}$, and $\Gamma_8^{(3)}$ – is predicted correctly for **[$Na_8Y_4ErF_{24}$]$^-$**. Figure S14 summarizes the energies and order of states for **[$Na_8Y_4ErF_{24}$]$^-$**, **[$Na_8Y_{10}ErF_{40}$]$^+$**, and $NaY_{0.98}Er_{0.02}F_4$.

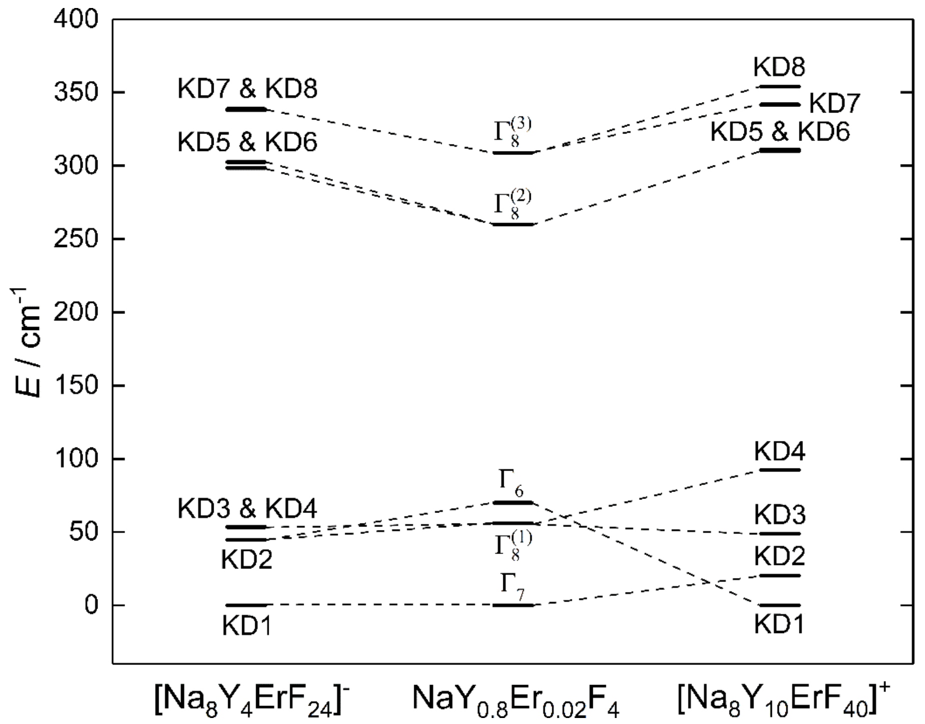

Supplementary Figure 14: Crystal field sublevel splitting. Splitting of the $^4I_{15/2}$ ground multiplet of Er^{3+} ion in **$[Na_8Y_4ErF_{24}]^-$**, $NaY_{0.98}Er_{0.02}F_4$ and **$[Na_8Y_{10}ErF_{40}]^+$**. For $NaY_{0.98}Er_{0.02}F_4$, the energies of states were adopted from the previously published data.[46]

Importantly, the rest of the results obtained for **$[Na_8Y_4ErF_{24}]^-$** also correlates well with the data reported in the EPR study of $NaY_{0.98}Er_{0.02}F_4$ (Figure 8, Table S24 and S31). As seen from Table S24, the ground KD of **$[Na_8Y_4ErF_{24}]^-$** is almost completely isotropic with the g_{avg} value of 6.79 ($g_x = 6.9$, $g_y = 6.91$, $g_z = 6.57$), in the perfect agreement with the EPR measurement of $NaY_{0.98}Er_{0.02}F_4$. The first excited state of **$[Na_8Y_4ErF_{24}]^-$** is the $\Gamma_8^{(1)}$ quartet state which consists of KD2 and KD4 as revealed by their compositions (Table S30) and averaged g-values (< 5.0; Table S24). The KD2 and KD4 are only split by 9 cm^{-1}, a result that is also in line with the EPR data (see above). Interestingly, the *ab initio* calculations predict the Γ_6 doublet state (KD3) to be anisotropic ($g_x = 7.77$, $g_y = 7.76$, $g_z = 2.19$) and lying between the Kramers's states (KD2 and KD4) of the $\Gamma_8^{(1)}$ quartet state (Figure 8 and Table S24). However, these two calculated results do not contradict the experimental findings and the KD3 can be assigned to the Γ_6 doublet state for three reasons. First, the value of the calculated g_{avg} is 5.90 for KD3 which is very close to the experimental g-value of 6.0 of the Γ_6 doublet state. Second, according to the EPR study of $NaY_{0.98}Er_{0.02}F_4$, the splitting of the $\Gamma_8^{(1)}$ quartet state is less than 28 cm^{-1} as mentioned above. Third, it has also been shown that when the Er^{3+} ions occupy the cubic symmetry sites in the Erbium-doped β-PbF_2 single-crystal, the Γ_6 doublet state can merge with the

$\Gamma_8^{(1)}$ quartet state, and it cannot be uniquely identified from the emission spectra of Erbium-doped β-PbF_2. In other words, the energy of the Γ_6 doublet state is very close to the energy of the $\Gamma_8^{(1)}$ quartet state in the Erbium-doped β-PbF_2.[50] The highest lying KD pairs, KD5 & KD6 and KD7 & KD8, can be assigned to the $\Gamma_8^{(2)}$ and $\Gamma_8^{(2)}$ quartet states, respectively, according the their energies, composition of the wavefunctions and *g*-values (Figure S14, Table S24 and S30). Considering all above mentioned, it can be concluded that the **$[Na_8Y_4ErF_{24}]^-$** fragment describes well the electronic structure of the investigated NPs and, thus, we only report its results in the main text.

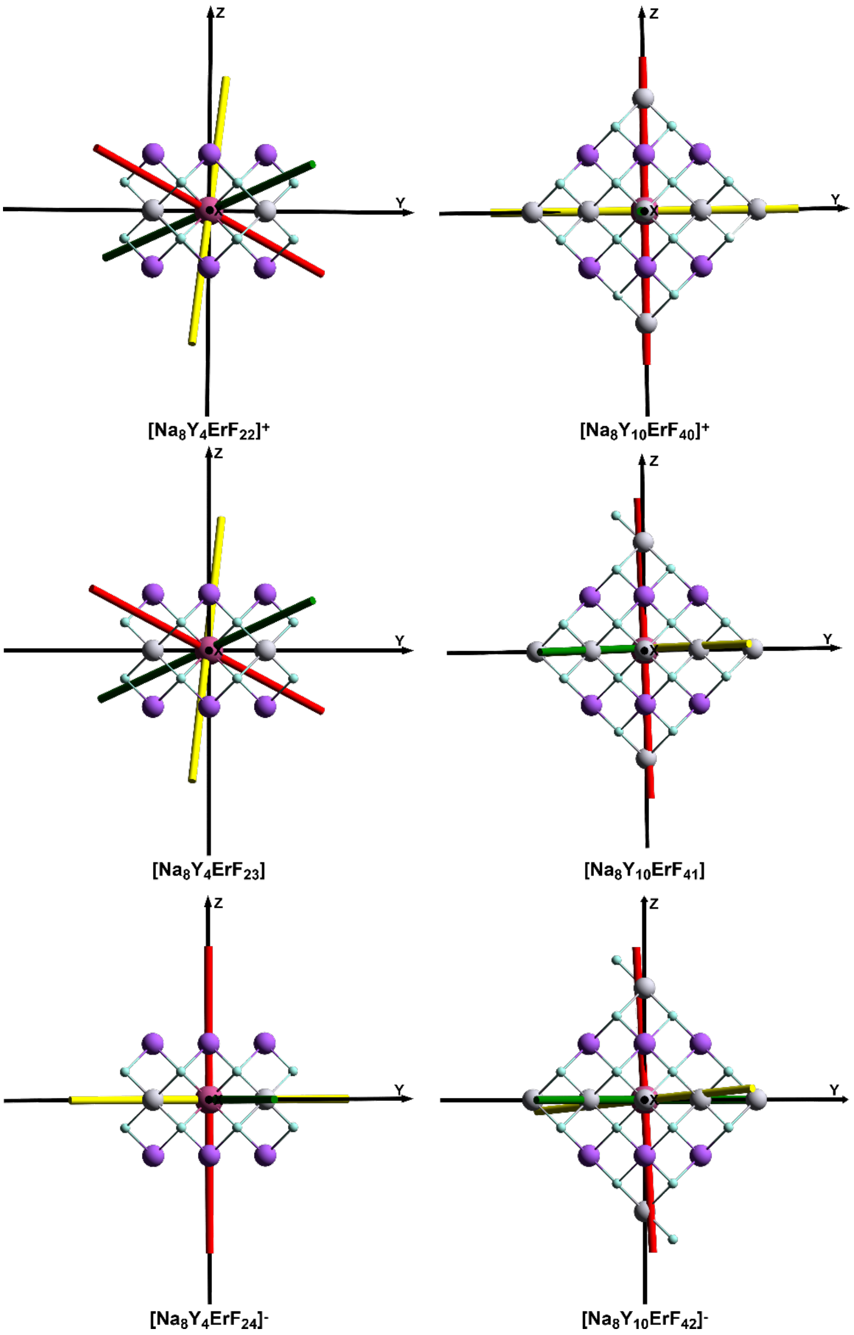

Supplementary Figure 15: Orientation of the main magnetic axes. Orientation of the main magnetic axes – X_m (green), Y_m, (yellow) and Z_m (red) – of the ground Kramers doublet of Er^{3+} ion in **$[Na_8Y_4ErF_{22}]^+$**, **$[Na_8Y_4ErF_{23}]$**, **$[Na_8Y_4ErF_{24}]^-$**, **$[Na_8Y_{10}ErF_{40}]^+$**, **$[Na_8Y_{10}ErF_{41}]$**, and **$[Na_8Y_{10}ErF_{42}]^-$**. Due to the low axial magnetic anisotropy of fragments **$[Na_8Y_4ErF_{24}]^-$**, **$[Na_8Y_{10}ErF_{40}]^+$**, **$[Na_8Y_{10}ErF_{41}]$**, and **$[Na_8Y_{10}ErF_{42}]^-$**, the direction of X_m and Y_m, axes are illustrated along with the quantization axis (Z_m) that is usually the main magnetization axis in strongly axial systems in which $g_z > g_x$ and g_y. The Z_m of **$[Na_8Y_{10}ErF_{40}]^+$** lie along the z-axis and its X_m and Y_m define the xy-plane. All fragments are visualized from the same viewing direction.

Supplementary Table 22: Calculated energies and ***g***-tensors of the eight lowest Kramers doublets arising from the crystal-field splitting of the ground $^4I_{15/2}$ multiplet of Er^{3+} ion as well as averaged g-values (g_{avg}) for **$[Na_8Y_4ErF_{22}]^+$**.

State	*E* / cm^{-1}	g_x	g_y	g_z	g_{avg}
**KD1**	0.00	2.84	3.52	12.89	6.42
**KD2**	51.19	0.10	3.67	8.89	4.22
**KD3**	68.83	0.28	2.83	11.65	4.92
**KD4**	79.49	1.22	4.16	12.08	5.82
**KD5**	272.37	0.13	0.19	16.29	5.54
**KD6**	330.43	1.33	4.03	11.81	5.73
**KD7**	352.75	0.14	4.05	9.56	4.58
**KD8**	374.59	1.26	2.83	13.56	5.88

Supplementary Table 23: Calculated energies and ***g***-tensors of the eight lowest Kramers doublets arising from the crystal-field splitting of the ground $^4I_{15/2}$ multiplet of Er^{3+} ion as well as averaged g-values (g_{avg}) for **$[Na_8Y_4ErF_{23}]$**.

State	*E* / cm^{-1}	g_x	g_y	g_z	g_{avg}
**KD1**	0.00	4.48	5.09	10.46	6.67
**KD2**	46.30	0.30	2.72	6.46	3.16
**KD3**	55.66	0.83	2.26	12.02	5.04
**KD4**	63.52	1.16	3.24	12.69	5.70
**KD5**	285.84	0.20	0.34	14.97	5.17
**KD6**	313.91	8.47	6.20	2.49	5.72
**KD7**	338.63	6.64	5.06	1.06	4.25
**KD8**	352.85	2.12	3.89	11.54	5.85

Supplementary Table 24: Calculated energies and ***g***-tensors of the eight lowest Kramers doublets arising from the crystal-field splitting of the ground $^4I_{15/2}$ multiplet of Er^{3+} ion as well as averaged g-values (g_{avg}) for **$[Na_8Y_4ErF_{24}]^-$**. The eigenstates of **$[Na_8Y_4ErF_{24}]^-$** are also given in Bethe notation.

State		*E* / cm^{-1}	g_x	g_y	g_z	g_{avg}
**KD1**	Γ_7	0.00	6.91	6.91	6.57	6.79
**KD2**	$\Gamma_8^{(1)}$	44.82	1.23	1.23	11.81	4.76
**KD3**	Γ_6	53.36	7.77	7.76	2.19	5.90
**KD4**	$\Gamma_8^{(1)}$	53.70	6.19	6.19	0.95	4.44
**KD5**	$\Gamma_8^{(2)}$	298.51	6.54	6.54	4.07	5.71
**KD6**		302.66	0.92	0.92	10.20	4.02
**KD7**	$\Gamma_8^{(3)}$	337.98	0.35	0.35	10.56	3.75
**KD8**		338.85	7.25	7.24	3.24	5.91

Supplementary Table 25: Calculated energies and ***g***-tensors of the eight lowest Kramers doublets arising from the crystal-field splitting of the ground $^4I_{15/2}$ multiplet of Er^{3+} ion as well as averaged g-values (g_{avg}) for **$[Na_8Y_{10}ErF_{40}]^+$**. The eigenstates of **$[Na_8Y_{10}ErF_{40}]^+$** are also given in Bethe notation.

State		***E* / cm^{-1}**	g_x	g_y	g_z	g_{avg}
**KD1**	Γ_6	0.00	8.10	8.10	2.05	6.08
**KD2**	Γ_7	20.44	9.06	9.06	1.64	6.59
**KD3**	$\Gamma_8^{(1)}$	48.95	7.55	7.55	3.26	6.12
**KD4**		92.34	0.07	0.07	14.74	4.96
**KD5**	$\Gamma_8^{(2)}$	309.94	0.47	0.47	10.31	3.75
**KD6**		311.36	6.41	6.40	4.25	5.69
**KD7**	$\Gamma_8^{(3)}$	341.85	6.94	6.94	3.73	5.87
**KD8**		354.22	0.02	0.02	11.56	3.87

Supplementary Table 26: Calculated energies and ***g***-tensors of the eight lowest Kramers doublets arising from the crystal-field splitting of the ground $^4I_{15/2}$ multiplet of Er^{3+} ion as well as averaged g-values (g_{avg}) for **$[Na_8Y_{10}ErF_{41}]$**.

**State**	***E* / cm^{-1}**	g_x	g_y	g_z	g_{avg}
**KD1**	0.00	8.45	7.77	1.69	5.97
**KD2**	18.44	9.38	8.75	1.63	6.59
**KD3**	49.31	7.60	7.54	3.60	6.24
**KD4**	96.42	0.05	0.07	14.64	4.92
**KD5**	309.72	1.66	2.71	10.30	4.89
**KD6**	312.93	3.49	4.09	8.33	5.30
**KD7**	342.26	7.06	6.68	3.89	5.88
**KD8**	356.11	0.16	0.17	11.73	4.02

Supplementary Table 27: Calculated energies and ***g***-tensors of the eight lowest Kramers doublets arising from the crystal-field splitting of the ground $^4I_{15/2}$ multiplet of Er^{3+} ion as well as averaged g-values (g_{avg}) for **$[Na_8Y_{10}ErF_{42}]^-$**.

**State**	***E* / cm^{-1}**	g_x	g_y	g_z	g_{avg}
**KD1**	0.00	8.88	7.35	1.33	5.85
**KD2**	16.47	9.80	8.33	1.60	6.58
**KD3**	50.01	7.60	7.53	3.95	6.36
**KD4**	101.09	0.04	0.07	14.53	4.88
**KD5**	309.54	1.99	3.06	10.47	5.17
**KD6**	315.08	3.43	3.67	8.06	5.05
**KD7**	343.12	7.11	6.41	4.15	5.89
**KD8**	358.97	0.17	0.29	12.05	4.17

Supplementary Table 28: Weights of M_J components (given in percents) in the wavefunctions (wf) of the ground multiplet J = 15/2 of **[Na$_8$Y$_4$ErF$_{22}$]$^+$**.

	**KD1**		**KD2**		**KD3**		**KD4**		**KD5**		**KD6**		**KD7**		**KD8**	
**M$_J$**	wf1	wf2	wf3	wf4	wf5	wf6	wf7	wf8	wf9	wf10	wf11	wf12	wf13	wf14	wf15	wf16
**-15/2**	16.8	1.7	0.9	1.5	1.9	0.1	1.3	0.8	1.2	69.6	0.7	0.0	0.1	2.9	0.0	0.4
**-13/2**	44.6	6.3	4.6	16.9	0.7	2.5	0.3	0.1	0.3	18.3	3.4	1.3	0.1	0.6	0.0	0.0
**-11/2**	12.2	1.1	15.8	27.8	5.6	4.2	4.5	2.8	0.5	1.5	10.6	0.0	4.8	8.0	0.5	0.1
**-9/2**	1.3	1.4	1.4	9.4	7.5	14.0	13.3	5.9	0.2	0.5	7.1	8.6	15.6	4.7	9.1	0.1
**-7/2**	0.1	0.2	3.5	1.4	5.9	6.0	21.3	2.6	1.1	0.5	17.2	3.4	2.5	12.7	11.5	10.1
**-5/2**	1.5	0.6	1.6	1.4	4.7	10.4	5.9	13.5	1.3	1.7	10.4	0.8	13.4	1.7	15.9	15.1
**-3/2**	6.6	0.7	2.1	0.1	19.7	6.3	6.5	0.2	0.1	0.3	5.4	18.0	13.3	1.5	0.7	18.5
**-1/2**	0.8	4.2	7.9	3.7	5.4	5.1	15.0	5.8	1.6	1.3	7.7	5.5	16.6	1.5	7.3	10.7
**1/2**	4.2	0.8	3.7	7.9	5.1	5.4	5.8	15.0	1.3	1.6	5.5	7.7	1.5	16.6	10.7	7.3
**3/2**	0.7	6.6	0.1	2.1	6.3	19.7	0.2	6.5	0.3	0.1	18.0	5.4	1.5	13.3	18.5	0.7
**5/2**	0.6	1.5	1.4	1.6	10.4	4.7	13.5	5.9	1.7	1.3	0.8	10.4	1.7	13.4	15.1	15.9
**7/2**	0.2	0.1	1.4	3.5	6.0	5.9	2.6	21.3	0.5	1.1	3.4	17.2	12.7	2.5	10.1	11.5
**9/2**	1.4	1.3	9.4	1.4	14.0	7.5	5.9	13.3	0.5	0.2	8.6	7.1	4.7	15.6	0.1	9.1
**11/2**	1.1	12.2	27.8	15.8	4.2	5.6	2.8	4.5	1.5	0.5	0.0	10.6	8.0	4.8	0.1	0.5
**13/2**	6.3	44.6	16.9	4.6	2.5	0.7	0.1	0.3	18.3	0.3	1.3	3.4	0.6	0.1	0.0	0.0
**15/2**	1.7	16.8	1.5	0.9	0.1	1.9	0.8	1.3	69.6	1.2	0.0	0.7	2.9	0.1	0.4	0.0

Supplementary Table 29: Weights of M_J components (given in percents) in the wavefunctions (wf) of the ground multiplet J = 15/2 of **[Na$_8$Y$_4$ErF$_{23}$]**.

	**KD1**		**KD2**		**KD3**		**KD4**		**KD5**		**KD6**		**KD7**		**KD8**	
**M$_J$**	wf1	wf2	wf3	wf4	wf5	wf6	wf7	wf8	wf9	wf10	wf11	wf12	wf13	wf14	wf15	wf16
**-15/2**	14.1	0.0	0.6	5.3	1.5	0.4	1.5	0.1	60.7	5.1	0.9	0.1	5.9	3.2	0.2	0.5
**-13/2**	39.0	0.4	1.8	26.3	1.4	5.5	0.8	0.2	15.5	0.9	3.1	2.0	1.2	1.8	0.0	0.1
**-11/2**	12.2	2.4	21.8	6.0	8.1	9.4	9.6	3.3	2.0	0.5	6.0	1.1	5.5	11.3	0.8	0.1
**-9/2**	3.4	1.7	10.1	1.2	11.6	4.7	13.6	7.4	1.4	0.1	5.8	7.0	3.3	15.3	12.6	1.1
**-7/2**	0.5	0.7	1.8	5.0	2.9	9.6	11.3	9.8	2.3	0.8	13.6	4.5	7.4	6.7	20.6	2.4
**-5/2**	4.1	0.3	0.7	1.4	8.8	9.1	15.7	0.6	4.0	1.0	4.9	5.4	0.1	14.0	10.7	19.2
**-3/2**	10.2	2.0	1.6	1.5	14.1	3.8	6.2	4.1	0.6	0.2	11.8	16.5	4.2	4.3	0.3	18.6
**-1/2**	2.5	6.6	15.0	0.0	2.9	6.1	14.1	1.8	2.7	2.3	11.1	6.4	0.4	15.3	0.8	11.9
**1/2**	6.6	2.5	0.0	15.0	6.1	2.9	1.8	14.1	2.3	2.7	6.4	11.1	15.3	0.4	11.9	0.8
**3/2**	2.0	10.2	1.5	1.6	3.8	14.1	4.1	6.2	0.2	0.6	16.5	11.8	4.3	4.2	18.6	0.3
**5/2**	0.3	4.1	1.4	0.7	9.1	8.8	0.6	15.7	1.0	4.0	5.4	4.9	14.0	0.1	19.2	10.7
**7/2**	0.7	0.5	5.0	1.8	9.6	2.9	9.8	11.3	0.8	2.3	4.5	13.6	6.7	7.4	2.4	20.6
**9/2**	1.7	3.4	1.2	10.1	4.7	11.6	7.4	13.6	0.1	1.4	7.0	5.8	15.3	3.3	1.1	12.6
**11/2**	2.4	12.2	6.0	21.8	9.4	8.1	3.3	9.6	0.5	2.0	1.1	6.0	11.3	5.5	0.1	0.8
**13/2**	0.4	39.0	26.3	1.8	5.5	1.4	0.2	0.8	0.9	15.5	2.0	3.1	1.8	1.2	0.1	0.0
**15/2**	0.0	14.1	5.3	0.6	0.4	1.5	0.1	1.5	5.1	60.7	0.1	0.9	3.2	5.9	0.5	0.2

Supplementary Table 30: Weights of M_J components (given in percents) in the wavefunctions (wf) of the ground multiplet J = 15/2 of $\mathbf{[Na_8Y_4ErF_{24}]^-}$.

	KD1		KD2		KD3		KD4		KD5		KD6		KD7		KD8	
**M_J**	wf1	wf2	wf3	wf4	wf5	wf6	wf7	wf8	wf9	wf10	wf11	wf12	wf13	wf14	wf15	wf16
**-15/2**	0.0	0.0	0.3	59.0	15.8	1.9	0.0	0.0	0.0	0.0	0.0	0.0	22.6	0.4	0.0	0.0
**-13/2**	38.5	0.7	0.0	0.0	0.0	0.0	21.4	0.1	0.7	14.6	0.0	0.0	0.0	0.0	14.1	10.0
**-11/2**	0.1	5.6	0.0	0.0	0.0	0.0	0.1	21.9	50.7	2.3	0.0	0.0	0.0	0.0	8.0	11.3
**-9/2**	0.0	0.0	1.2	0.0	0.5	3.9	0.0	0.0	0.0	0.0	86.7	7.7	0.0	0.1	0.0	0.0
**-7/2**	0.0	0.0	0.1	18.6	4.0	0.5	0.0	0.0	0.0	0.0	0.0	0.1	75.5	1.3	0.0	0.0
**-5/2**	32.8	0.6	0.0	0.0	0.0	0.0	6.7	0.0	0.8	16.6	0.0	0.0	0.0	0.0	24.9	17.6
**-3/2**	0.4	21.3	0.0	0.0	0.0	0.0	0.3	49.5	13.8	0.6	0.0	0.0	0.0	0.0	5.8	8.2
**-1/2**	0.0	0.0	20.8	0.1	7.8	65.8	0.0	0.0	0.0	0.0	5.0	0.4	0.0	0.1	0.0	0.0
**1/2**	0.0	0.0	0.1	20.8	65.8	7.8	0.0	0.0	0.0	0.0	0.4	5.0	0.1	0.0	0.0	0.0
**3/2**	21.3	0.4	0.0	0.0	0.0	0.0	49.5	0.3	0.6	13.8	0.0	0.0	0.0	0.0	8.2	5.8
**5/2**	0.6	32.8	0.0	0.0	0.0	0.0	0.0	6.7	16.6	0.8	0.0	0.0	0.0	0.0	17.6	24.9
**7/2**	0.0	0.0	18.6	0.1	0.5	4.0	0.0	0.0	0.0	0.0	0.1	0.0	1.3	75.5	0.0	0.0
**9/2**	0.0	0.0	0.0	1.2	3.9	0.5	0.0	0.0	0.0	0.0	7.7	86.7	0.1	0.0	0.0	0.0
**11/2**	5.6	0.1	0.0	0.0	0.0	0.0	21.9	0.1	2.3	50.7	0.0	0.0	0.0	0.0	11.3	8.0
**13/2**	0.7	38.5	0.0	0.0	0.0	0.0	0.1	21.4	14.6	0.7	0.0	0.0	0.0	0.0	10.0	14.1
**15/2**	0.0	0.0	59.0	0.3	1.9	15.8	0.0	0.0	0.0	0.0	0.0	0.0	0.4	22.6	0.0	0.0

Supplementary Table 31: Weights of M_J components (given in percents) in the wavefunctions (wf) of the ground multiplet J = 15/2 of $\mathbf{[Na_8Y_{10}ErF_{40}]^+}$.

	KD1		KD2		KD3		KD4		KD5		KD6		KD7		KD8	
**M_J**	wf1	wf2	wf3	wf4	wf5	wf6	wf7	wf8	wf9	wf10	wf11	wf12	wf13	wf14	wf15	wf16
**-15/2**	0.0	0.0	0.0	0.0	0.0	0.0	66.4	0.3	0.0	0.0	0.0	0.0	0.0	0.0	0.0	33.3
**-13/2**	20.3	0.5	0.0	0.0	27.4	4.3	0.0	0.0	0.0	0.0	16.8	0.4	30.0	0.2	0.0	0.0
**-11/2**	0.2	9.6	0.0	0.0	1.7	10.9	0.0	0.0	0.0	0.0	1.4	54.7	0.1	21.4	0.0	0.0
**-9/2**	0.0	0.0	4.6	0.0	0.0	0.0	0.0	0.0	95.1	0.3	0.0	0.0	0.0	0.0	0.0	0.0
**-7/2**	0.0	0.0	0.0	0.0	0.0	0.0	33.2	0.1	0.0	0.0	0.0	0.0	0.0	0.0	0.0	66.7
**-5/2**	26.5	0.6	0.0	0.0	18.1	2.8	0.0	0.0	0.0	0.0	14.2	0.4	37.2	0.2	0.0	0.0
**-3/2**	1.0	41.2	0.0	0.0	4.7	30.2	0.0	0.0	0.0	0.0	0.3	11.8	0.1	10.7	0.0	0.0
**-1/2**	0.0	0.0	94.8	0.5	0.0	0.0	0.0	0.1	4.6	0.0	0.0	0.0	0.0	0.0	0.0	0.0
**1/2**	0.0	0.0	0.5	94.8	0.0	0.0	0.1	0.0	0.0	4.6	0.0	0.0	0.0	0.0	0.0	0.0
**3/2**	41.2	1.0	0.0	0.0	30.2	4.7	0.0	0.0	0.0	0.0	11.8	0.3	10.7	0.1	0.0	0.0
**5/2**	0.6	26.5	0.0	0.0	2.8	18.1	0.0	0.0	0.0	0.0	0.4	14.2	0.2	37.2	0.0	0.0
**7/2**	0.0	0.0	0.0	0.0	0.0	0.0	0.1	33.2	0.0	0.0	0.0	0.0	0.0	0.0	66.7	0.0
**9/2**	0.0	0.0	0.0	4.6	0.0	0.0	0.0	0.0	0.3	95.1	0.0	0.0	0.0	0.0	0.0	0.0
**11/2**	9.6	0.2	0.0	0.0	10.9	1.7	0.0	0.0	0.0	0.0	54.7	1.4	21.4	0.1	0.0	0.0
**13/2**	0.5	20.3	0.0	0.0	4.3	27.4	0.0	0.0	0.0	0.0	0.4	16.8	0.2	30.0	0.0	0.0
**15/2**	0.0	0.0	0.0	0.0	0.0	0.0	0.3	66.4	0.0	0.0	0.0	0.0	0.0	0.0	33.3	0.0

Supplementary Table 32: Weights of M_J components (given in percents) in the wavefunctions (wf) of the ground multiplet J = 15/2 of **[$Na_8Y_{10}ErF_{41}$]**.

	KD1		KD2		KD3		KD4		KD5		KD6		KD7		KD8	
**M_J**	wf1	wf2	wf3	wf4	wf5	wf6	wf7	wf8	wf9	wf10	wf11	wf12	wf13	wf14	wf15	wf16
**-15/2**	0.0	0.1	0.0	0.0	0.0	0.1	1.3	64.1	0.0	0.0	0.0	0.0	0.0	0.0	0.0	34.3
**-13/2**	1.4	17.8	0.0	0.0	0.0	32.2	0.0	0.2	1.4	1.8	1.3	12.8	13.2	17.9	0.0	0.0
**-11/2**	9.9	0.2	0.0	0.0	11.8	0.1	0.0	0.0	3.4	2.7	44.4	6.2	13.7	7.4	0.1	0.1
**-9/2**	0.1	0.0	0.9	3.5	0.2	0.0	0.0	0.6	71.7	8.5	7.1	5.6	0.0	0.1	0.0	1.8
**-7/2**	0.0	0.4	0.1	0.0	0.0	0.3	0.7	32.6	1.8	0.1	0.5	0.0	0.0	0.1	0.1	63.3
**-5/2**	0.5	25.3	0.0	0.1	0.3	22.3	0.0	0.5	0.8	1.5	1.5	9.9	15.3	21.7	0.0	0.3
**-3/2**	41.3	1.4	0.8	1.4	32.4	0.0	0.0	0.0	1.0	0.9	9.9	0.5	4.9	5.5	0.0	0.0
**-1/2**	1.2	0.2	31.7	61.5	0.5	0.0	0.0	0.0	3.4	1.0	0.1	0.1	0.1	0.1	0.0	0.0
**1/2**	0.2	1.2	61.5	31.7	0.0	0.5	0.0	0.0	1.0	3.4	0.1	0.1	0.1	0.1	0.0	0.0
**3/2**	1.4	41.3	1.4	0.8	0.0	32.4	0.0	0.0	0.9	1.0	0.5	9.9	5.5	4.9	0.0	0.0
**5/2**	25.3	0.5	0.1	0.0	22.3	0.3	0.5	0.0	1.5	0.8	9.9	1.5	21.7	15.3	0.3	0.0
**7/2**	0.4	0.0	0.0	0.1	0.3	0.0	32.6	0.7	0.1	1.8	0.0	0.5	0.1	0.0	63.3	0.1
**9/2**	0.0	0.1	3.5	0.9	0.0	0.2	0.6	0.0	8.5	71.7	5.6	7.1	0.1	0.0	1.8	0.0
**11/2**	0.2	9.9	0.0	0.0	0.1	11.8	0.0	0.0	2.7	3.4	6.2	44.4	7.4	13.7	0.1	0.1
**13/2**	17.8	1.4	0.0	0.0	32.2	0.0	0.2	0.0	1.8	1.4	12.8	1.3	17.9	13.2	0.0	0.0
**15/2**	0.1	0.0	0.0	0.0	0.1	0.0	64.1	1.3	0.0	0.0	0.0	0.0	0.0	0.0	34.3	0.0

Supplementary Table 33: Weights of M_J components (given in percents) in the wavefunctions (wf) of the ground multiplet J = 15/2 of **[$Na_8Y_{10}ErF_{42}$]$^-$**.

	KD1		KD2		KD3		KD4		KD5		KD6		KD7		KD8	
**M_J**	wf1	wf2	wf3	wf4	wf5	wf6	wf7	wf8	wf9	wf10	wf11	wf12	wf13	wf14	wf15	wf16
**-15/2**	0.2	0.0	0.0	0.0	0.1	0.2	63.3	0.2	0.1	0.1	0.0	0.1	0.0	0.1	0.2	35.3
**-13/2**	16.7	0.3	0.0	0.1	12.2	20.0	0.8	0.0	3.5	0.5	3.4	9.6	14.5	18.2	0.0	0.1
**-11/2**	0.3	10.2	0.1	0.2	5.0	6.1	0.0	0.0	0.9	4.7	42.3	9.8	8.2	11.6	0.2	0.6
**-9/2**	0.1	0.7	0.2	3.6	0.4	0.1	2.1	0.0	12.9	60.8	12.0	0.3	0.2	0.3	0.1	6.2
**-7/2**	1.5	0.0	0.1	0.4	0.5	0.8	31.4	0.1	1.7	4.8	1.7	0.6	0.2	0.0	0.3	55.9
**-5/2**	22.9	1.0	0.0	0.4	14.7	9.7	1.8	0.0	2.2	0.3	1.9	7.7	16.5	19.7	0.0	1.1
**-3/2**	0.8	39.6	2.4	6.2	15.6	12.6	0.0	0.0	0.4	1.9	6.3	4.2	7.5	2.4	0.1	0.1
**-1/2**	0.1	5.6	23.4	63.0	1.5	0.4	0.0	0.0	0.6	4.6	0.1	0.0	0.3	0.3	0.0	0.0
**1/2**	5.6	0.1	63.0	23.4	0.4	1.5	0.0	0.0	4.6	0.6	0.0	0.1	0.3	0.3	0.0	0.0
**3/2**	39.6	0.8	6.2	2.4	12.6	15.6	0.0	0.0	1.9	0.4	4.2	6.3	2.4	7.5	0.1	0.1
**5/2**	1.0	22.9	0.4	0.0	9.7	14.7	0.0	1.8	0.3	2.2	7.7	1.9	19.7	16.5	1.1	0.0
**7/2**	0.0	1.5	0.4	0.1	0.8	0.5	0.1	31.4	4.8	1.7	0.6	1.7	0.0	0.2	55.9	0.3
**9/2**	0.7	0.1	3.6	0.2	0.1	0.4	0.0	2.1	60.8	12.9	0.3	12.0	0.3	0.2	6.2	0.1
**11/2**	10.2	0.3	0.2	0.1	6.1	5.0	0.0	0.0	4.7	0.9	9.8	42.3	11.6	8.2	0.6	0.2
**13/2**	0.3	16.7	0.1	0.0	20.0	12.2	0.0	0.8	0.5	3.5	9.6	3.4	18.2	14.5	0.1	0.0
**15/2**	0.0	0.2	0.0	0.0	0.2	0.1	0.2	63.3	0.1	0.1	0.1	0.0	0.1	0.0	35.3	0.2

Supplementary Table 34: Calculated *ab initio* crystal-field parameters B_{kq} (cm^{-1}) presented in the Iwahara–Chibotaru notation[51, 52] for **$[Na_8Y_4ErF_{22}]^+$**.

k	q	Re|B_{kq}|	Im|B_{kq}|	|B_{kq}|
2	0	52.601	0.000	52.601
2	1	0.000	0.000	0.000
2	2	0.000	0.000	0.000
4	0	-101.064	0.000	101.064
4	1	0.000	0.000	0.000
4	2	0.000	0.000	0.000
4	3	0.000	0.000	0.000
4	4	-56.014	5.559	56.289
6	0	43.154	0.000	43.154
6	1	0.000	0.000	0.000
6	2	0.000	0.000	0.000
6	3	0.000	0.000	0.000
6	4	-72.525	7.198	72.882
6	5	0.000	0.000	0.000
6	6	0.000	0.000	0.000
8	0	-0.014	0.000	0.014
8	1	0.000	0.000	0.000
8	2	0.000	0.000	0.000
8	3	0.000	0.000	0.000
8	4	-0.052	0.005	0.052
8	5	0.000	0.000	0.000
8	6	0.000	0.000	0.000
8	7	0.000	0.000	0.000
8	8	-0.002	0.000	0.002
10	0	-0.006	0.000	0.006
10	1	0.000	0.000	0.000
10	2	0.000	0.000	0.000
10	3	0.000	0.000	0.000
10	4	0.031	-0.003	0.031
10	5	0.000	0.000	0.000
10	6	0.000	0.000	0.000
10	7	0.000	0.000	0.000
10	8	-0.006	0.001	0.006
10	9	0.000	0.000	0.000
10	10	0.000	0.000	0.000
12	0	0.004	0.000	0.004
12	1	0.000	0.000	0.000
12	2	0.000	0.000	0.000
12	3	0.000	0.000	0.000
12	4	-0.005	0.000	0.005
12	5	0.000	0.000	0.000
12	6	0.000	0.000	0.000
12	7	0.000	0.000	0.000
12	8	0.005	-0.001	0.005

12	9	0.000	0.000	0.000
12	10	0.000	0.000	0.000
12	11	0.000	0.000	0.000
12	12	0.000	0.000	0.000
14	0	0.000	0.000	0.000
14	1	0.000	0.000	0.000
14	2	0.000	0.000	0.000
14	3	0.000	0.000	0.000
14	4	0.000	0.000	0.000
14	5	0.000	0.000	0.000
14	6	0.000	0.000	0.000
14	7	0.000	0.000	0.000
14	8	0.000	0.000	0.000
14	9	0.000	0.000	0.000
14	10	0.000	0.000	0.000
14	11	0.000	0.000	0.000
14	12	0.000	0.000	0.000
14	13	0.000	0.000	0.000
14	14	0.000	0.000	0.000

Supplementary Table 35: Calculated *ab initio* crystal-field parameters B_{kq} (cm^{-1}) presented in the Iwahara–Chibotaru notation[51, 52] for **[$Na_8Y_4ErF_{23}$]**.

k	q	Re|B_{kq}|	Im|B_{kq}|	|B_{kq}|
2	0	-28.32	0.00	28.32
2	1	0.92	2.95	3.09
2	2	4.06	2.25	4.64
4	0	42.03	0.00	42.03
4	1	27.56	-16.43	32.09
4	2	-28.45	-7.32	29.37
4	3	50.62	-41.06	65.18
4	4	3.15	-27.18	27.36
6	0	33.59	0.00	33.59
6	1	22.17	-33.22	39.94
6	2	25.62	-15.71	30.05
6	3	-21.58	10.87	24.17
6	4	0.26	22.16	22.16
6	5	-9.45	16.38	18.91
6	6	9.04	-39.34	40.36
8	0	0.01	0.00	0.01
8	1	-0.07	0.09	0.11
8	2	0.01	0.12	0.12
8	3	0.07	-0.01	0.07
8	4	0.01	-0.02	0.02
8	5	-0.03	-0.03	0.04
8	6	-0.01	0.01	0.02
8	7	0.01	-0.01	0.02
8	8	0.00	0.01	0.01
10	0	-0.01	0.00	0.01
10	1	0.04	0.01	0.05
10	2	-0.03	-0.02	0.04
10	3	-0.01	-0.01	0.02
10	4	0.00	0.03	0.03
10	5	0.01	0.02	0.02
10	6	0.01	0.01	0.01
10	7	-0.01	0.00	0.01
10	8	0.00	-0.01	0.01
10	9	0.00	0.00	0.00
10	10	0.00	0.00	0.00
12	0	-0.01	0.00	0.01
12	1	-0.01	-0.01	0.01
12	2	0.00	0.00	0.00
12	3	0.00	0.00	0.00
12	4	0.00	0.00	0.00
12	5	0.00	0.00	0.00
12	6	0.00	0.00	0.00
12	7	0.00	0.00	0.00
12	8	0.00	0.00	0.00

12	9	0.00	0.00	0.00
12	10	0.00	0.00	0.00
12	11	0.00	0.00	0.00
12	12	0.00	0.00	0.00
14	0	0.00	0.00	0.00
14	1	0.00	0.00	0.00
14	2	0.00	0.00	0.00
14	3	0.00	0.00	0.00
14	4	0.00	0.00	0.00
14	5	0.00	0.00	0.00
14	6	0.00	0.00	0.00
14	7	0.00	0.00	0.00
14	8	0.00	0.00	0.00
14	9	0.00	0.00	0.00
14	10	0.00	0.00	0.00
14	11	0.00	0.00	0.00
14	12	0.00	0.00	0.00
14	13	0.00	0.00	0.00
14	14	0.00	0.00	0.00

Supplementary Table 36: Calculated *ab initio* crystal-field parameters B_{kq} (cm^{-1}) presented in the Iwahara–Chibotaru notation[51, 52] for **[$Na_8Y_4ErF_{24}$]$^-$**.

k	q	Re|B_{kq}|	Im|B_{kq}|	|B_{kq}|
2	0	-3.69	0.00	3.69
2	1	0.00	0.00	0.00
2	2	0.00	0.00	0.00
4	0	-100.82	0.00	100.82
4	1	0.00	0.00	0.00
4	2	0.00	0.00	0.00
4	3	0.00	0.00	0.00
4	4	10.07	-51.09	52.08
6	0	39.74	0.00	39.74
6	1	0.00	0.00	0.00
6	2	0.00	0.00	0.00
6	3	0.00	0.00	0.00
6	4	14.15	-71.79	73.17
6	5	0.00	0.00	0.00
6	6	0.00	0.00	0.00
8	0	-0.03	0.00	0.03
8	1	0.00	0.00	0.00
8	2	0.00	0.00	0.00
8	3	0.00	0.00	0.00
8	4	0.00	-0.01	0.01
8	5	0.00	0.00	0.00
8	6	0.00	0.00	0.00
8	7	0.00	0.00	0.00
8	8	0.00	0.00	0.00
10	0	-0.01	0.00	0.01
10	1	0.00	0.00	0.00
10	2	0.00	0.00	0.00
10	3	0.00	0.00	0.00
10	4	-0.01	0.03	0.03
10	5	0.00	0.00	0.00
10	6	0.00	0.00	0.00
10	7	0.00	0.00	0.00
10	8	0.01	0.00	0.01
10	9	0.00	0.00	0.00
10	10	0.00	0.00	0.00
12	0	0.00	0.00	0.00
12	1	0.00	0.00	0.00
12	2	0.00	0.00	0.00
12	3	0.00	0.00	0.00
12	4	0.00	0.00	0.00
12	5	0.00	0.00	0.00
12	6	0.00	0.00	0.00
12	7	0.00	0.00	0.00
12	8	0.00	0.00	0.00

12	9	0.00	0.00	0.00
12	10	0.00	0.00	0.00
12	11	0.00	0.00	0.00
12	12	0.00	0.00	0.00
14	0	0.00	0.00	0.00
14	1	0.00	0.00	0.00
14	2	0.00	0.00	0.00
14	3	0.00	0.00	0.00
14	4	0.00	0.00	0.00
14	5	0.00	0.00	0.00
14	6	0.00	0.00	0.00
14	7	0.00	0.00	0.00
14	8	0.00	0.00	0.00
14	9	0.00	0.00	0.00
14	10	0.00	0.00	0.00
14	11	0.00	0.00	0.00
14	12	0.00	0.00	0.00
14	13	0.00	0.00	0.00
14	14	0.00	0.00	0.00

Supplementary Table 37: Calculated *ab initio* crystal-field parameters B_{kq} (cm^{-1}) presented in the Iwahara–Chibotaru notation[51, 52] for **$[Na_8Y_{10}ErF_{40}]^+$**.

k	q	Re|B_{kq}|	Im|B_{kq}|	|B_{kq}|
2	0	52.601	0.000	52.601
2	1	0.000	0.000	0.000
2	2	0.000	0.000	0.000
4	0	-101.064	0.000	101.064
4	1	0.000	0.000	0.000
4	2	0.000	0.000	0.000
4	3	0.000	0.000	0.000
4	4	-56.014	5.559	56.289
6	0	43.154	0.000	43.154
6	1	0.000	0.000	0.000
6	2	0.000	0.000	0.000
6	3	0.000	0.000	0.000
6	4	-72.525	7.198	72.882
6	5	0.000	0.000	0.000
6	6	0.000	0.000	0.000
8	0	-0.014	0.000	0.014
8	1	0.000	0.000	0.000
8	2	0.000	0.000	0.000
8	3	0.000	0.000	0.000
8	4	-0.052	0.005	0.052
8	5	0.000	0.000	0.000
8	6	0.000	0.000	0.000
8	7	0.000	0.000	0.000
8	8	-0.002	0.000	0.002
10	0	-0.006	0.000	0.006
10	1	0.000	0.000	0.000
10	2	0.000	0.000	0.000
10	3	0.000	0.000	0.000
10	4	0.031	-0.003	0.031
10	5	0.000	0.000	0.000
10	6	0.000	0.000	0.000
10	7	0.000	0.000	0.000
10	8	-0.006	0.001	0.006
10	9	0.000	0.000	0.000
10	10	0.000	0.000	0.000
12	0	0.004	0.000	0.004
12	1	0.000	0.000	0.000
12	2	0.000	0.000	0.000
12	3	0.000	0.000	0.000
12	4	-0.005	0.000	0.005
12	5	0.000	0.000	0.000
12	6	0.000	0.000	0.000
12	7	0.000	0.000	0.000
12	8	0.005	-0.001	0.005

12	9	0.000	0.000	0.000
12	10	0.000	0.000	0.000
12	11	0.000	0.000	0.000
12	12	0.000	0.000	0.000
14	0	0.000	0.000	0.000
14	1	0.000	0.000	0.000
14	2	0.000	0.000	0.000
14	3	0.000	0.000	0.000
14	4	0.000	0.000	0.000
14	5	0.000	0.000	0.000
14	6	0.000	0.000	0.000
14	7	0.000	0.000	0.000
14	8	0.000	0.000	0.000
14	9	0.000	0.000	0.000
14	10	0.000	0.000	0.000
14	11	0.000	0.000	0.000
14	12	0.000	0.000	0.000
14	13	0.000	0.000	0.000
14	14	0.000	0.000	0.000

Supplementary Table 38: Calculated *ab initio* crystal-field parameters B_{kq} (cm^{-1}) presented in the Iwahara–Chibotaru notation[51, 52] for **[$Na_8Y_{10}ErF_{41}$]**.

k	q	Re|B_{kq}|	Im|B_{kq}|	|B_{kq}|
2	0	57.42	0.00	57.42
2	1	0.00	-0.45	0.45
2	2	-0.63	0.00	0.63
4	0	-100.04	0.00	100.04
4	1	0.00	8.46	8.46
4	2	0.14	0.00	0.14
4	3	0.00	-3.28	3.28
4	4	56.26	0.01	56.26
6	0	42.74	0.00	42.74
6	1	0.00	-5.27	5.27
6	2	-0.72	0.00	0.72
6	3	0.00	-7.60	7.60
6	4	72.07	0.01	72.07
6	5	0.00	-6.38	6.38
6	6	-0.21	0.00	0.21
8	0	-0.01	0.00	0.01
8	1	0.00	0.00	0.00
8	2	0.00	0.00	0.00
8	3	0.00	-0.01	0.01
8	4	0.05	0.00	0.05
8	5	0.00	-0.03	0.03
8	6	0.00	0.00	0.00
8	7	0.00	0.00	0.00
8	8	0.00	0.00	0.00
10	0	0.00	0.00	0.00
10	1	0.00	0.01	0.01
10	2	0.00	0.00	0.00
10	3	0.00	0.01	0.01
10	4	-0.03	0.00	0.03
10	5	0.00	0.00	0.00
10	6	0.00	0.00	0.00
10	7	0.00	0.00	0.00
10	8	-0.01	0.00	0.01
10	9	0.00	0.00	0.00
10	10	0.00	0.00	0.00
12	0	0.00	0.00	0.00
12	1	0.00	0.00	0.00
12	2	0.00	0.00	0.00
12	3	0.00	0.00	0.00
12	4	0.00	0.00	0.00
12	5	0.00	0.00	0.00
12	6	0.00	0.00	0.00
12	7	0.00	0.00	0.00
12	8	0.00	0.00	0.00

12	9	0.00	0.00	0.00
12	10	0.00	0.00	0.00
12	11	0.00	0.00	0.00
12	12	0.00	0.00	0.00
14	0	0.00	0.00	0.00
14	1	0.00	0.00	0.00
14	2	0.00	0.00	0.00
14	3	0.00	0.00	0.00
14	4	0.00	0.00	0.00
14	5	0.00	0.00	0.00
14	6	0.00	0.00	0.00
14	7	0.00	0.00	0.00
14	8	0.00	0.00	0.00
14	9	0.00	0.00	0.00
14	10	0.00	0.00	0.00
14	11	0.00	0.00	0.00
14	12	0.00	0.00	0.00
14	13	0.00	0.00	0.00
14	14	0.00	0.00	0.00

Supplementary Table 39: Calculated *ab initio* crystal-field parameters B_{kq} (cm^{-1}) presented in the Iwahara–Chibotaru notation[51, 52] for $[Na_8Y_{10}ErF_{42}]^-$.

k	q	Re|B_{kq}|	Im|B_{kq}|	|B_{kq}|
2	0	62.83	0.00	62.83
2	1	0.00	-1.18	1.18
2	2	-1.21	0.00	1.21
4	0	-97.77	0.00	97.77
4	1	0.00	16.49	16.49
4	2	0.67	0.00	0.67
4	3	0.00	-6.47	6.47
4	4	56.04	0.00	56.04
6	0	41.20	0.00	41.20
6	1	0.00	-10.14	10.14
6	2	-2.83	0.00	2.83
6	3	0.00	-14.67	14.67
6	4	70.03	0.00	70.03
6	5	0.00	-12.42	12.42
6	6	-0.79	0.00	0.79
8	0	-0.01	0.00	0.01
8	1	0.00	-0.01	0.01
8	2	0.00	0.00	0.00
8	3	0.00	-0.02	0.02
8	4	0.04	0.00	0.04
8	5	0.00	-0.06	0.06
8	6	0.00	0.00	0.00
8	7	0.00	0.00	0.00
8	8	0.00	0.00	0.00
10	0	0.00	0.00	0.00
10	1	0.00	0.01	0.01
10	2	0.01	0.00	0.01
10	3	0.00	0.02	0.02
10	4	-0.03	0.00	0.03
10	5	0.00	0.00	0.00
10	6	0.00	0.00	0.00
10	7	0.00	0.00	0.00
10	8	-0.01	0.00	0.01
10	9	0.00	0.00	0.00
10	10	0.00	0.00	0.00
12	0	0.00	0.00	0.00
12	1	0.00	0.00	0.00
12	2	0.00	0.00	0.00
12	3	0.00	0.00	0.00
12	4	0.00	0.00	0.00
12	5	0.00	0.00	0.00
12	6	0.00	0.00	0.00
12	7	0.00	0.00	0.00
12	8	0.00	0.00	0.00

12	9	0.00	0.00	0.00
12	10	0.00	0.00	0.00
12	11	0.00	0.00	0.00
12	12	0.00	0.00	0.00
14	0	0.00	0.00	0.00
14	1	0.00	0.00	0.00
14	2	0.00	0.00	0.00
14	3	0.00	0.00	0.00
14	4	0.00	0.00	0.00
14	5	0.00	0.00	0.00
14	6	0.00	0.00	0.00
14	7	0.00	0.00	0.00
14	8	0.00	0.00	0.00
14	9	0.00	0.00	0.00
14	10	0.00	0.00	0.00
14	11	0.00	0.00	0.00
14	12	0.00	0.00	0.00
14	13	0.00	0.00	0.00
14	14	0.00	0.00	0.00

References

[1] Dovesi, R., et al. Quantum-mechanical condensed matter simulations with CRYSTAL. *WIREs Comput. Mol. Sci.* **8**, e1360 (2018).

[2] Togo, A., Chaput, L., Tadano, T. & Tanaka, I. Implementation strategies in phonopy and phono3py. *Phys.: Condens. Matter* **35**, 353001 (2023).

[3] Togo, A. First-principles phonon calculations with phonopy and phono3py. *J. Phys. Soc. Japan* **92**, 012001 (2023).

[4] Qin, X., et al. Suppression of defect-induced quenching via chemical potential tuning: A theoretical solution for enhancing lanthanide luminescence. *J. Phys. Chem. C.* **123**, 11151-11161 (2019).

[5] Perdew, J. P., Burke, K. & Ernzerhof, M. Generalized gradient approximation made simple. *Phys. Rev. Lett.* **77**, 3865 (1996).

[6] Peintinger, M. F., Oliveira, D. V. & Bredow, T. Consistent Gaussian basis sets of triple-zeta valence with polarization quality for solid-state calculations. *J. Comput. Chem.* **34**, 451-459 (2013).

[7] Laun, J., Oliveira, D. V. & Bredow, T. Consistent gaussian basis sets of double- and triple-zeta valence with polarization quality of the fifth period for solid-state calculations. *J. Comput. Chem.* **39**, 1285-1290 (2018).

[8] https://crysplot.crystalsolutions.eu/

[9] Wang, Y., et al. A mixed-space approach to first-principles calculations of phonon frequencies for polar materials. *J. Phys.: Condens. Matter* **22**, 202201 (2010).

[10] Yang, D., et al. Controllable phase transformation and mid-infrared emission from Er^{3+}-doped hexagonal-/cubic-$NaYF_4$ nanocrystals. *Sci. Rep.* **6**, 29871 (2016).

[11] Dubey, C., et al. Impact of crystal structure on optical properties and temperature sensing behavior of $NaYF_4$:Yb^{3+}/Er^{3+} nanoparticles. *RSC Adv.* **13**, 20975-20983 (2023).

[12] Assaaoudi, H., Shan, G.-B., Dyck, N. & Demopoulos, G. P. Annealing-induced ultra-efficient NIR-to-VIS upconversion of nano-/micro-scale α and β $NaYF_4$:Er^{3+},Yb^{3+} crystals. *CrystEngComm.* **15**, 4739-4746 (2013).

[13] Yang, D., et al. Controllable phase transformation and mid-infrared emission from Er^{3+}-doped hexagonal-/cubic-$NaYF_4$ nanocrystals. *Sci. Rep.* **6**, 29871 (2016).

[14] Wang, Z., Li, X., Zhang, G., Luo, Y. & Jiang, J. Suppressing electron–phonon coupling through laser-induced phase transition. *ACS Appl. Mater. Interfaces* **9**, 23309-23313 (2017).

[15] Thokwane, P. & Mbule, P. Analysis of the $NaYF_4$:Yb^{3+}, Er^{3+} nanocrystals: up-conversion luminescence, crystal structure and morphology influenced by the dopant concentration and annealing temperature. *Bull. Mater. Sci.* **46**, 140 (2023).

[16] Wang, G., et al. Controlled synthesis and luminescence properties from cubic to hexagonal $NaYF_4:Ln^{3+}$ (Ln = Eu and Yb/Tm) microcrystals. *J. Alloys Compd.* **475**, 452-455 (2009).

[17] Renero-Lecuna, C., et al. Origin of the high upconversion green luminescence efficiency in β-$NaYF_4$:2%Er^{3+},20%Yb^{3+}. *Chem. Mater.* **23**, 3442-3448 (2011).

[18] Suyver, J. F., et al. Upconversion spectroscopy and properties of $NaYF_4$ doped with Er^{3+}, Tm^{3+} and/or Yb^{3+}. *J. Lumin.* **117**, 1-12 (2006).

[19] Chen, Q., Li, Z., Miao, B. & Ma, Q. Thermal, nonlinear, magnetic and faraday rotation properties of sol-gel diamagnetic glass/$NaYF_4$: Fe, Ho^{3+}: Role of magnetic ions. *J. Alloys. Compd.* **858**, 157631 (2021).

[20] Tuyen, V. T., et al. Controllable structural and optical properties of $NaYF_4$:Tm, Yb microparticles by Yb^{3+} doping for anti-counterfeiting. *RSC Adv.* **13**, 19317-19324 (2023).

[21] Luo, X. & Akimoto, K. Upconversion properties in hexagonal-phase $NaYF_4:Er^{3+}/NaYF_4$ nanocrystals by off-resonant excitation. *Appl. Surf. Sci.* **273**, 257-260 (2013).

[22] Briganti, M., Santanni, F., Tesi, L., Totti, F., Sessoli, R. & Lunghi, A. A complete *Ab initio* view of Orbach and Raman spin-lattice relaxation in a dysprosium coordination compound. *J. Am. Chem. Soc.* **143**, 13633-13645 (2021).

[23] Wang, Li., et al. A new cubic phase for a $NaYF_4$ host matrix offering high upconversion luminescence efficiency. *Adv. Mater.* **27**, 5528-5533 (2015).

[24] Wang, Z., Li, X., Zhang, G., Luo, Y. & Jiang, J. Suppressing electron–phonon coupling through laser-induced phase transition. *ACS Appl. Mater. Interfaces* **9**, 23309-23313 (2017).

[25] Kumar, K., et al. Ratiometric Raman and luminescent thermometers constructed from dysprosium thiocyanidometallate molecular magnets. *Adv. Opt. Mater.* **10**, 2201675 (2022).

[26] Kumar, K., et al. Detection of sub-terahertz Raman response and nonlinear optical effects for luminescent Yb(III) complexes. *Adv. Opt. Mater.* **10**, 2101721 (2022).

[27] Fdez. Galván, I. et al. OpenMolcas: From source code to insight. *J. Chem. Theory Comput.* **15**, 5925-5964 (2019).

[28] Roy, D. M. & Roy, R. Controlled massively defective crystalline solutions with the fluorite structure. *J. Electrochem. Soc.* **111**, 421 (1964).

[29] Roos, B. O., et al. New relativistic atomic natural orbital basis sets for lanthanide atoms with applications to the Ce diatom and LuF_3. *J. Phys. Chem. A* **112**, 11431-11435 (2008).

[30] Roos, B. O., Lindh, R., Malmqvist, Per-Ake, Veryazov, V. & Widmark, P.-O. Main group atoms and dimers studied with a new relativistic ANO basis set. *J. Phys. Chem. A* **108**, 2851-2858 (2004).

[31] Kutzelnigg, W. & Liu, W. Quasirelativistic theory equivalent to fully relativistic theory. *J. Chem. Phys.* **123**, 241102 (2005).

[32] Filatov, M. Comment on "Quasirelativistic theory equivalent to fully relativistic theory" [J. Chem. Phys. 123, 241102 (2005)]. *J. Chem. Phys.* **125**, 107101 (2006).

[33] Peng, D. & Reiher, M. Exact decoupling of the relativistic Fock operator. *Theor. Chem. Acc.* **131**, 1081 (2012).

[34] Roos, B. O., Lindh, R., Malmqvist, P. Å., Veryazov, V. & Widmark, P.-O. *Multiconfigurational Quantum Chemistry*; John Wiley & Sons, Inc.: Hoboken, NJ, USA, **2016**.

[35] Roos, B. O., Taylor, P. R. & Sigbahn, P. E. M. A complete active space SCF method (CASSCF) using a density matrix formulated super-CI approach. *Chem. Phys.* **48**, 157-173 (1980).

[36] Siegbahn, P., Heiberg, A., Roos, B. & Levy, B. A comparison of the Super-CI and the Newton-Raphson scheme in the complete active space SCF method. *Phys. Scr.* **21**, 323-327 (1980).

[37] Malmqvist, P. Å., Roos, B. O. & Schimmelpfennig, B. The restricted active space (RAS) state interaction approach with spin–orbit coupling. *Chem. Phys. Lett.* **357**, 230-240 (2002).

[38] Ungur, L. & Chibotaru, L. F. Ab Initio crystal field for lanthanides. *Chem. - A Eur. J.* **23**, 3708-3718 (2017).

[39] Ungur, L., Thewissen, M., Costes, J.-P., Wernsdorfer, W. & Chibotaru, L. F. Interplay of strongly anisotropic metal ions in magnetic blocking of complexes. *Inorg. Chem.* **52**, 6328-6337 (2013).

[40] Chibotaru, L. F. & Ungur, L. Ab Initio calculation of anisotropic magnetic properties of complexes. I. Unique definition of pseudospin hamiltonians and their derivation. *J. Chem. Phys.* **137**, 064112 (2012).

[41] Chibotaru, L. F., Ungur, L. & Soncini, A. The origin of nonmagnetic Kramers doublets in the ground state of dysprosium triangles: Evidence for a toroidal magnetic moment. *Angew. Chem. Int. Ed.* **47**, 4126-4129 (2008).

[42] Ungur, L., Van den Heuvel, W. & Chibotaru, L. F. *Ab Initio* investigation of the non-collinear magnetic structure and the lowest magnetic excitations in dysprosium triangles. *New J. Chem.* **33**, 1224-1230 (2009).

[43] Grebenyuk, D., et al. A family of lanthanide hydroxo carboxylates with 1D polymeric topology and Ln_4 butterfly core exhibits switchable supramolecular arrangement. *Inorg. Chem.* **60**, 8049-8061 (2021).

[44] Topor, A., et al. Design of Fe^{III}–Ln^{III} binuclear complexes using compartmental ligands: Synthesis, crystal structures, magnetic properties, and *ab initio* analysis. *J. Mater. Chem. C.* **9**, 10912-10926 (2021).

[45] Yang, Q., Ungur, L., Wernsdorfer, W. & Tang, J. Toroidal magnetic moments in Tb_4 squares. *Inorg. Chem. Front.* **9**, 784-791 (2022).

[46] García-Flores, A. F., et al. Crystal-field stark effect on the upconversion light emission spectrum of $\alpha - NaYF_4$ nanoparticles doped with Dy^{3+}, Er^{3+}, or Yb^{3+}. *Phys. Rev. B.* **106**, 125427 (2022).

[47] Lea, K. R., Leask, M. J. M. & Wolf, W. P. The raising of angular momentum degeneracy of f-electron terms by cubic crystal fields. *J. Phys. Chem. Solids.* **23**, 1381-1405 (1962).
[48] Pake, G. E. & Estle, T. L. *The Physical Principles of Electron Paramagnetic Resonance*; Benjamin: London, England, **1973**.
[49] Dziesiaty, J., et al. Non-cubic Er centres in ZnSe studied by electron paramagnetic resonance and optical analysis *J. Phys.: Condens. Matter*. **7**, 4271 (1995).
[50] Dantelle, G., Mortier, M. & Vivien, D. EPR and optical studies of erbium-doped β-PbF_2 single-crystals and nanocrystals in transparent glass–ceramics. *Phys. Chem. Chem. Phys.* **9**, 5591-5598 (2007).
[51] Iwahara, N. & Chibotaru, L. F. Exchange interaction between *J* multiplets. *Phys. Rev. B.* **91**, 174438 (2015).
[52] Iwahara, N., Ungur, L. & Chibotaru, L. F. *J*-pseudospin states and the crystal field of cubic systems. *Phys. Rev. B.* **98**, 054436 (2018).
