# Supplementary material for: Confining single Er3+ ions in sub-3 nm NaYF4 nanoparticles to induce slow relaxation of the magnetisation
Source: Nat Commun. 2024 Apr 25;15:3498. doi: 10.1038/s41467-024-47682-x (PMC11045814; doi:10.1038/s41467-024-47682-x)
Supplement: Supplementary file 2 — Description of Additional Supplementary Files [file 41467_2024_47682_MOESM2_ESM.pdf]

**Description of Additional Supplementary Files**

Supplementary Data 1: includes data to visualize the vibrational spectrum of α-$NaYF_4$. It is a output file generated by CRYSTAL17 program and it can be opened with CRYSPLOT, which is a web platform to visualize physical and chemical properties of molecules, polymers, surfaces and crystalline solids (http://crysplot.crystalsolutions.eu/). To open the output file, choose the top menu called “Make a plot” on the main webpage of CRYSPLOT and from the menu select the option “Vibrational spectra & animations”. Once the option “Vibrational spectra & animations” is selected new webpage opens where the output file can be uploaded, and the vibrational spectrum can be visualized. More information about the visualization of vibration spectra is given on the webpage of CRYSPLOT.

Supplementary Data 2: includes data to visualize the vibrational spectrum of α-$NaY^{167.259}F_4$. It is a output file generated by CRYSTAL17 program and it can be opened with CRYSPLOT, which is a web platform to visualize physical and chemical properties of molecules, polymers, surfaces and crystalline solids (http://crysplot.crystalsolutions.eu/). To open the output file, choose the top menu called “Make a plot” on the main webpage of CRYSPLOT and from the menu select the option “Vibrational spectra & animations”. Once the option “Vibrational spectra & animations” is selected new webpage opens where the output file can be uploaded, and the vibrational spectrum can be visualized. More information about the visualization of vibration spectra is given on the webpage of CRYSPLOT.
